# Permutation Tests Based on the Copula‐Graphic Estimator and Their Use for Survival Tree Construction

**DOI:** 10.1002/sim.70483

**Published:** 2026-03-16

**Authors:** Pauline Baur, Markus Pauly, Takeshi Emura

**Affiliations:** ^1^ Department of Statistics TU Dortmund University Dortmund Germany; ^2^ Research Center Trustworthy Data Science and Security University Alliance Ruhr Dortmund Germany; ^3^ School of Informatics and Data Science Hiroshima University Hiroshima Japan

**Keywords:** classification tree, copula, dependent censoring

## Abstract

Survival trees are popular alternatives to Cox or Aalen regression models that offer both modeling flexibility and graphical interpretability. This paper introduces a new algorithm for survival trees that relaxes the assumption of independent censoring. To this end, we use the copula‐graphic estimator to estimate survival functions. This allows us to flexibly specify shape and strength of the dependence of survival and censoring times within survival trees. For splitting, we present a permutation test for the null hypothesis of equal survival. Our test statistic consists of the integrated absolute distance of the groups' copula‐graphic estimators. A first simulation study shows a good type I error and power behavior of the new test. We thereby assess simulation settings of various group sizes, censoring percentages, and grades of dependence generated by Clayton and Frank copulas. Using this test as a splitting criterion, a second simulation study studies the performance of the resulting trees and compares it with that of the usual logrank‐based tree. Lastly, the tree algorithm is applied to real‐world clinical trial data.

**Abbreviations**
CGE: copula‐graphic estimator
PBC: primary biliary cholangitis

## 1 Introduction

In survival analysis, censoring is a common phenomenon, occurring, for example, when participants are lost to follow‐up in a clinical trial. Typically, both censoring times and survival times are random [1]. Many common survival analysis methods are derived under the assumption of independent censoring and survival times [1, 2]. However, this assumption may not always be realistic, potentially introducing bias into the survival analysis [3, 4]. For example, Klein and Moeschberger (1987) [5] explain this issue for the case of the Kaplan‐Meier estimator.

Dependent censoring can arise, for instance, when study dropouts occur due to adverse events or lack of improvement from the study medication [6], since a patient's health status affects both these events and the expected survival time. In case of a positive dependency between survival and censoring times, subjects censored at a time t have a smaller expected survival compared to subjects censored at a time greater than t. Consequently, statistical methods assuming independent censoring can overestimate the survival time. The opposite is true for negative dependency [7]. This bias is especially problematic when two groups with varying censoring proportions are compared. One such scenario is a clinical placebo‐controlled trial, where the verum group has a higher dropout rate due to study‐drug‐related adverse events. If the independent censoring assumption is violated, applying biased survival time estimation can lead to overly optimistic survival estimates for the verum group [3, 8]. Hence, survival analysis methods that can model dependent censoring are often necessary.

Copulas are a common tool in such methods. They specify the joint distribution of random variables [9, chapter 2], in our case event and censoring times. Furthermore, copula models can be used to analyze the bias introduced by false independent censoring assumptions, as Emura and Chen (2016) [4] show for univariate feature selection using Cox‐regression. Zheng and Klein (1995) [10] introduce a copula‐based estimator for survival distributions, the copula‐graphic estimator. It can be considered an extension of the Kaplan–Meier estimator that incorporates dependence using a pre‐specified copula model. Many applications of this estimator exist: Lo and Wilke (2010) [11] extend the copula‐graphic estimator for data with more than two competing risks using the class of Archimedean copulas. Huang and Zhang (2008) [3] apply the work of Zheng and Klein to a regression setting. They model marginal competing risks using Cox proportional hazard models, while the joint distribution is modeled on an assumed copula. Many further applications of the copula‐graphic estimator in survival regression settings have been explored, including frequentist approaches [12, 13] as well as Bayesian methods [6].

The present paper uses the copula‐graphic estimator to derive nonparametric survival trees that allow for dependent censoring. Traditional classification and regression trees, which both are nonlinear regression models, were introduced by Breiman et al. (1984) [14]. Various extensions of their work to survival analysis under the independent censoring assumption have been developed [15]. In the present paper, we extend the idea behind conditional inference trees [16] and the work of Emura et al. (2023) [17] to the setting of dependent censoring. We will construct trees of binary splits on single covariates using p‐values of significance tests for survival difference as a splitting criterion. We will modify existing tree algorithms, such as logrank trees [18], by using a significance test that does not assume independent censoring. In doing so, we present a new survival analysis method that is straightforward to implement, easy to interpret and free from assumptions of parametric regression models.

To this end, we propose a permutation test with the integrated, absolute distance of the copula‐graphic estimators of two groups as a test statistic for the null hypothesis of equal survival distributions, assuming equal censoring distributions across groups. Permutation tests are a common tool in survival analysis [16, 19, 20] and similar approaches to ours already exist: Pepe and Fleming (1989) [21] introduce a class of Kaplan‐Meier estimator based statistics. They extend their statistic by various weighting functions that reduce the impact of observations towards the end of a study when only few events are observed. Moradian et al. (2017) [22] use a statistic of absolute distances of Kaplan‐Meier estimators as a splitting criterion in a survival forest. They later extend their work using the copula‐graphic estimator to create a survival forest that can account for dependent censoring [23]. As detailed in Section 2.3, we propose a standardized version of the distance proposed by Modarian et al. (2019) [23]. In addition, our proposed approach is based on p‐values, which provide a statistically more meaningful measure than the distance itself.

The rest of the paper will be structured as follows: Section 2 will review the copula‐graphic estimator and survival trees and then propose a survival tree algorithm for dependently censored survival data. Section 3 and 4 will assess the algorithm's performance in two simulation studies. Lastly, in Section 5 we will apply the survival tree algorithm to real‐world data from the Mayo Clinic Primary Biliary Cholangitis clinical trial. Section 6 concludes the paper with a discussion.

## 2 Methods

### 2.1 Notation

We consider right‐censored survival data for n subjects. Event times are modeled by non‐negative random variables $T_{i}$, corresponding censoring times by non‐negative $C_{i}$

$$T_{i}∼F,\;C_{i}∼G,\;i=1,\ldots n$$

with continuous, strictly increasing distribution functions F and G, respectively. For each subject i, only $X_{i}=\min(T_{i},C_{i})$ and the censoring status $\Delta_{i}=1(X_{i}=T_{i})$ can be observed with 1() being the indicator function. The $X_{i}$s are assumed to be independent, identically distributed random variables. In addition, we observe p covariates, which are a realization of the random vector $Z_{i}={\left(Z_{i1},\ldots ,Z_{ip}\right)}^{⊤}$. Thus, the observed dataset is $\left\{(x_{i},\delta_{i},z_{i});i=1,\ldots ,n\right\}$ with lowercase letters denoting the realizations of the respective random variables. Throughout, vectors are denoted using bold font. The probability of subject i surviving past a time t>0 is given by the survival function $S_{T}(t)=Pr(T_{i}>t)=1-F(t)$, which is continuous and strictly decreasing [24, chapter 2]. The corresponding censoring function is defined analogously as $S_{C}(t)=Pr(C_{i}>t)=1-G(t)$. Let T and C denote independent copies of $T_{i}$ and $C_{i}$, respectively.

### 2.2 Copula‐Graphic Estimator

In the following, T and C are not independent, instead their dependency will be modeled using bivariate copulas. These will be defined based on survival functions, rather than cumulative distribution functions, to fit our survival setting. The general properties of copulas remain valid [25, chapter 3]. The joint survival function of T and C is

$$Pr(T>t,C>s)=𝒞\left(S_{T}(t),S_{C}(s)\right),\;s,t>0,$$

with 𝒞 being the copula. A feasible copula function 𝒞 has to fulfill 𝒞(u,0)=𝒞(0,v)=0, 𝒞(u,1)=u and 𝒞(1,v)=v for every u,v∈[0,1]. Furthermore, we require 𝒞 to yield a probability mass on every rectangle in ${\left[0,1\right]}^{2}$, by ensuring $𝒞(u_{2},v_{2})-𝒞(u_{2},v_{1})-𝒞(u_{1},v_{2})+𝒞(u_{1},v_{1})\geq 0$ for all $u_{1},u_{2},v_{1},v_{2}\in [0,1]$ with $u_{1}\leq u_{2}$ and $v_{1}\leq v_{2}$ [9, chapter 2].

The scope of this paper will be restricted to the class of Archimedean copulas, which have a closed‐form expression and thus are convenient to work with. Archimedean copulas are generated by a function φ via

(1) $$𝒞\left(S_{T}(t),S_{C}(s)\right)=\varphi^{-1}\left(\varphi \left(S_{T}(t)\right)+\varphi \left(S_{C}(s)\right)\right);[\text{25, chapter 3}]$$

for φ:[0,1]→[0,∞] being continuous and strictly decreasing to φ(1)=0 with pseudo‐inverse

$$\varphi^{-1}(u)=\left\{\begin{array}{ll} \varphi^{-1}(u),\;\; & 0\leq u\leq \varphi (0)\\ 0,\;\; & \varphi (0)\leq u\leq \infty . \end{array}\right.$$

Equation (1) defines a copula, if φ is convex. One example for an Archimedean copula is the Clayton copula, which is generated by $\varphi (u)=\left(u^{-\theta }-1\right)/\theta$ for a parameter θ∈[−1,∞)∖{0} [9, chapter 4].

Copulas provide an easy‐to‐interpret way of modeling the dependence structure, specifically the concordance of T and C. On a realization level, a concordant pair of observations $\left(t_{1},c_{1}\right)$ and $\left(t_{2},c_{2}\right)$ fulfills $(t_{1}-t_{2})(c_{1}-c_{2})>0$; a discordant one $(t_{1}-t_{2})(c_{1}-c_{2})<0$. On the population level, the scale‐invariant Kendall's τ measures association between i.i.d. vectors $\left(T_{1},C_{1}\right)$ and $\left(T_{2},C_{2}\right)$ with

$$\tau =Pr\left((T_{1}-T_{2})(C_{1}-C_{2})>0\right)-Pr\left((T_{1}-T_{2})(C_{1}-C_{2})<0\right),$$

which is the difference of the probability of concordance and the probability of discordance. For random variables $S_{T}(T)$ and $S_{C}(C)$ with dependence structure 𝒞, Kendall's τ can alternatively be calculated as

(2) $$\tau =4𝔼\left(𝒞\left(S_{T}(T),S_{C}(C)\right)\right)-1\overset{\left(*\right)}{=}1+4\overset{1}{\underset{0}{\int }}\frac{\varphi (t)}{\varphi^{\prime }(t)}dt$$

with the last equation (∗) holding true for Archimedean 𝒞 [9, chapter 5]. Thus, the association depends on the copula, but not the respective marginal distributions. For the Clayton copula, Equation (2) simplifies to τ=θ/(θ+2), yielding a straightforward way to specify the level of concordance [9, chapter 5]. Consequently, the Clayton copula's limiting case of θ→0 is the independence copula, which models independent event and censoring times as $𝒞\left(S_{T}(t),S_{C}(s)\right)=Pr(T>t)Pr(C>s)$. It can be generated from Equation (1) by choosing φ(u)=−log(u) [9, chapter 4].

As explained in the introduction, the well‐known Kaplan‐Meier estimator does not consider a dependency of survival and censoring times. For scenarios, where the independent censoring assumption is not realistic, Zheng and Klein (1995) [10] introduced an alternative estimator, the copula‐graphic estimator (CGE), which estimates the survival function under a known dependence structure described by a copula. Rivest and Wells (2001) [26] extend this work by deriving a closed‐form expression of the CGE for survival and censoring functions under the assumption of an Archimedean copula with twice‐differentiable generator φ. They start by requesting the naive estimate for the survival function $\hat{\pi }(t)=1/n\sum_{i=1}^{n}1(X_{i}>t)$ at time t>0 to be equal to the Archimedean copula structure from Equation (1) based on estimators of $S_{T}$ and $S_{C}$, rather than the theoretical survival and censoring function. This can be denoted as

$$\varphi^{-1}\left[\varphi (Ŝ(X_{i}))+\varphi (Ĉ(X_{i}))\right]=\hat{\pi }(X_{i}),\;i\in \{1,\ldots ,n\}$$

with Ŝ and Ĉ being the CGEs of the survival and censoring function, respectively. Solving this equation for Ŝ and considering n observations, yields the CGE of the survival function:

(3) $$\begin{array}{ll} Ŝ(t) & =\varphi^{-1}\left[-\underset{X_{i}\leq t,\delta_{i}=1}{\sum }\varphi (\hat{\pi }(X_{i}))-\varphi \left(\hat{\pi }(X_{i})-\frac{1}{n}\right)\right],\\ & \;0\leq t\leq \max(X_{1},\ldots X_{n}). \end{array}$$

It is a right‐continuous, decreasing step‐function. Ŝ(0) equals 1. Subsequently, there are negative jumps at each $x_{i}$ associated with an event ($\delta_{i}=1$). Using the generator of the independence copula φ(u)=−log(u) in Equation (3), the CGE Ŝ(t) equals the Kaplan‐Meier estimator for $t<\max(X_{1},\ldots X_{n})$ [10, 26].

For instance, under a Clayton copula, the CGE is given by: [12]

$$\begin{array}{ll} \hat{S}(t) & =\left(1-\sum_{X_{i}\leq t,\delta_{i}=1}\left({\left(\frac{\sum_{j=1}^{n}1\{X_{j}>X_{i}\}-1}{n}\right)}^{\theta }\right.\right.-{\left.\left.{\left(\frac{\sum_{j=1}^{n}1\{X_{j}>X_{i}\}}{n}\right)}^{\theta }\right)\right)}^{-\frac{1}{\theta }},\\ & \;0\leq t\leq \max(X_{1},\overset{\ldots }{,}X_{n}). \end{array}$$

**FIGURE B1:**
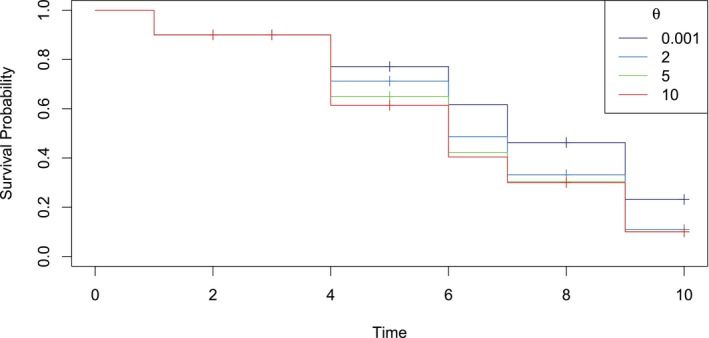
Copula‐graphic estimator with Clayton copula and varying dependency parameter θ for data $x={\left(1,2,3,4,5,6,7,8,9,10\right)}^{⊤}$ and $\delta ={\left(1,0,0,1,0,1,1,0,1,1\right)}^{⊤}$. Censorings are marked by a +‐symbol. See how increasing θ assumes a stronger dependency of event and censoring times and thus leads to a stronger influence of censorings on the jump size of the copula‐graphic estimator.

A visualization of the CGE and the influence of the assumed dependency through the copula model can be found in Figure B1 on page 95 in the Appendix B.

### 2.3 Proposed Test

In the following paragraphs, we propose a permutation test assessing differences in survival time distributions between two groups using a randomization technique for the exact control of the type I error rate. The notation introduced in Section 2.1 will be extended to cover a two‐sample problem. For group j∈{1,2}, the event and censoring time variables will be

$$T_{ji}∼F_{j},\;C_{ji}∼G_{j},\;j=1,2,\;i=1,\ldots ,n_{j},$$

with respective distributions $F_{j}$ and $G_{j}$. Censored data $X_{ji}=\min(T_{ji},C_{ji})$ and $\Delta_{ji}=1(X_{ji}=T_{ji})$ are adapted accordingly. The null and alternative hypotheses for a difference in survival distributions of the two samples are given by

(4) $$H_{0}:S_{T_{11}}=S_{T_{21}}\;\text{vs.}\;H_{1}:S_{T_{11}}\neq S_{T_{21}}.$$

The censoring distributions for both groups, $G_{1}$ and $G_{2}$, are assumed to be identical, such that the $X_{ij}$ are exchangeable under the null hypothesis. We will use the CGE for Archimedean copulas to construct a test statistic for a non‐parametric permutation test. The test statistic is similar to the one proposed by Moradian et al. (2019) [23], who used a similar statistic as a measure of prognostic difference between groups for determining optimal splits in a survival random forest. We slightly modify the statistic and will introduce a permutation test and corresponding splitting criterion.

Intuitively, the statistic is derived from the absolute difference of the CGEs of groups 1 and 2 and should increase with differing $S_{T_{11}}$ and $S_{T_{21}}$. For an observation vector $x={\left({x_{1}}^{⊤},{x_{2}}^{⊤}\right)}^{⊤}$ with observations in group j being $x_{j}={\left(\min(t_{j1},c_{j1}),\ldots ,\min(t_{jn_{j}},c_{jn_{j}})\right)}^{⊤}$ and censoring indicator vector $\delta ={\left({\delta_{1}}^{⊤},{\delta_{2}}^{⊤}\right)}^{⊤}$, the statistic is

(5) $$L_{1}(x,\delta )=\overset{\min(\max(x_{1}),\max(x_{2}))}{\underset{\min(x_{1},x_{2})}{\int }}\frac{\left|{Ŝ}_{T_{11}}(t)-{Ŝ}_{T_{21}}(t)\right|}{\min(\max(x_{1}),\max(x_{2}))}dt.$$

The normalization of the integrated difference by the observed time span $t^{*}=\min(\max(x_{1}),\max(x_{2}))$ is a modification compared to the statistic used by Moradian et al. (2019) [23]. This has two purposes: First, it makes the test statistic primarily reflect the magnitude of differences between groups during the common observation window rather than the overall study length. Second, truncation at $t^{*}$ limits the influence of a few extreme observations in the longer‐followed group that could otherwise inflate the CGE difference. The resulting statistic is a time‐normalized average CGE difference. This adjustment addresses situations where one group has a substantially shorter follow‐up with a rapidly declining CGE and a small maximum observed time, while the other group has longer observed survival times: Without normalization, the integral may be small solely due to the short integration range, even when the between‐group survival difference is large.

Since the theoretical probability distribution of $L_{1}$ is difficult to be derived analytically, we will evaluate it using a randomization approach.

To find the permutation distribution of $L_{1}$ and calculate corresponding p‐values, we consider the finite permutation group

$$𝒢=\left\{g:{\mathbb{R}}^{2\times n}\to {\mathbb{R}}^{2\times n},\left(\left(\begin{array}{l} x_{1}\\ \delta_{1} \end{array}\right),\ldots ,\left(\begin{array}{l} x_{n}\\ \delta_{n} \end{array}\right)\right)\mapsto \left(\left(\begin{array}{l} x_{\pi (1)}\\ \delta_{\pi (1)} \end{array}\right),\ldots ,\left(\begin{array}{l} x_{\pi (n)}\\ \delta_{\pi (n)} \end{array}\right)\right)\right\}$$

of size |𝒢|=n! for any permuting function π:{1,…,n}→{1,…,n}. Note that each observation's event time and censoring indicator are permuted jointly, ensuring that an observed event time always remains an event time and cannot be permuted into a censoring time and vice versa. Furthermore, the group sizes $n_{1}$ and $n_{2}$ stay the same.

Enabled by the assumption of exchangeability and equal censoring distributions across groups, the distribution of (X,Δ) with $X=\left(X_{11},\ldots ,X_{1n_{1}},X_{21},\ldots ,X_{2n_{2}}\right)$ is invariant to permutations in 𝒢 under the null hypothesis. In this case, the following test ψ based on statistic $L_{1}$ is an exact level α test for $H_{0}$ as in Equation (4), that is, E(ψ(X,Δ))=α: [27, chapter 17]

(6) $$\psi (x,\delta )=\left\{\begin{array}{ll} 1,\; & \text{if}\;L_{1}(x,\delta )>L_{1}^{\left(k\right)}(x,\delta )\\ a(x,\delta ),\; & \text{if}\;L_{1}(x,\delta )=L_{1}^{\left(k\right)}(x,\delta )\\ 0,\; & \text{if}\;L_{1}(x,\delta )<L_{1}^{\left(k\right)}(x,\delta ). \end{array}\right.$$

$L_{1}(x,\delta )$ is the test statistic on the observed data and critical value $L_{1}^{\left(k\right)}(x,\delta )$ is derived by calculating the test statistics for all |𝒢| permutations, ordering them to $L_{1}^{\left(1\right)}(x,\delta )\leq L_{1}^{\left(2\right)}(x,\delta )\leq \cdots \leq L_{1}^{\left(n!\right)}(x,\delta )$ and considering the k=(n!−⌊n!α⌋)th value. By setting randomization probability $a(x,\delta )=\left(\alpha n!-|\{j:L_{1}^{\left(j\right)}(x,\delta )>L_{1}^{\left(k\right)}(x,\delta )\}|\right)\cdot {\left(\left|\{j:L_{1}^{\left(j\right)}(x,\delta )=L_{1}^{\left(k\right)}(x,\delta )\}\right|\right)}^{-1},j\in \{1,\ldots n!\}$, we ensure that ψ is an exact level‐α‐test.

In most cases, a systematic calculation of all n! permutations would be computationally infeasible. Even for moderate sample sizes of $n_{1}=n_{2}=50$, we would already have to consider $100!=9.332622\times 10^{157}$ permutations. Exploiting the test statistic's symmetry reduces this to an effective number of n1+n2n1 distinct permutations, which still becomes infeasible quickly, for example, with 50+5050=1.008913×1029 for $n_{1}=n_{2}=50$. We therefore resort to $n_{perm}\leq n!$ random data permutations. We consider the observed data and $n_{perm}-1$ further permutations, since the observed group assignment has to be part of the group of considered permutations to obtain positive Monte Carlo p‐values. This yields a valid test, in the sense that under $H_{0}$ (exchangeability) the probability of seeing a p‐value as small as some p∈(0,1) is at most p. However, under the alternative hypothesis the test may be slightly less powerful than an exact (fully permuted) permutation test [28, chapter 3.5].

The resulting algorithm for its p‐value computation is given below:

ALGORITHM 1 **p‐value of introduced randomization test.**

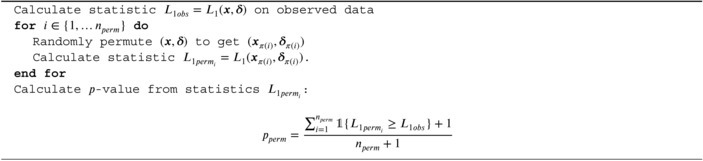

### 2.4 Survival Trees

We will construct survival trees using recursive partitioning, which repeatedly splits the covariate space into two disjoint sub‐spaces, yielding increasingly homogeneous survival outcomes within and heterogeneous outcomes between groups. A basic method of tree building uses binary splits based on a single covariate at a time [15]. Each node partitions the data into child nodes $\left\{i:z_{ij}\leq q\right\}$ and $\left\{i:z_{ij}>q\right\}$ based on the jth covariable and some cutoff value q, for i∈{1,…,n} and j∈{1,…,p}.

Each split is chosen to maximize the prognostic survival difference between the resulting groups. Ciampi et al. (1986) [18] use a logrank test as a measure for prognostic difference. Performing a grid search over all covariates and feasible cutoff values, logrank test p‐values are calculated for all possible splits. The optimal split is chosen as the split that maximizes the test statistic among all significant tests to some pre‐defined p‐value threshold. Nodes are split until no feasible split with a p‐value smaller than the threshold can be found. Emura et al. (2023) [17] generally adapt this approach, but select the split that minimizes the p‐value after variance stabilization. We will follow their approach, but use the test introduced in Section 2.3 instead of a logrank test, in order to account for possibly dependent censoring. Moradian et al. (2019) [23] already used a similar statistic to construct survival trees, however, their statistic was not evaluated within a statistical test and they constructed a random forest rather than a single tree.

Typically, median survival time and Kaplan–Meier estimator of the resulting terminal nodes are reported. We will supplement this by the CGE. For ideal interpretation of the tree, we aim to order the terminal nodes by survival prognosis from left to right.

To do so, the test statistic in (5) is calculated without absolute values, resulting in

$${\tilde{L}}_{1}(x,\delta )=\int_{\min(x_{1},x_{2})}^{\min(\max(x_{1}),\max(x_{2}))}\frac{{Ŝ}_{T_{11}}(t)-{Ŝ}_{T_{21}}(t)}{\min(\max(x_{1}),\max(x_{2}))}\;dt.$$

A positive value of ${\tilde{L}}_{1}$ indicates a longer survival in group 1. Based on the sign of ${\tilde{L}}_{1}$, group 1 is assigned to either the left or the right child node, ensuring that subjects with better survival prognosis always move to the right side of the tree. This yields the following tree algorithm (Algorithm 2):

ALGORITHM 2 **Construction of survival tree.**

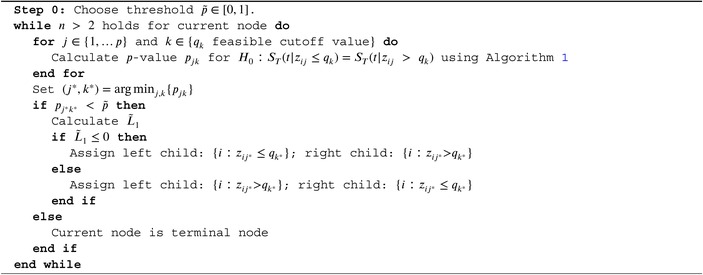

### 2.5 Choice of Copula Model and Dependency Parameter

Since the true marginal distribution of survival and censoring times is not identifiable from an observed competing risk dataset [29], additional assumptions have to be made prior to estimation. One assumption providing identifiability is the copula assumption [10] introduced above. Therefore selecting a sensible copula generator φ and dependence τ in Equation (3) will be a crucial and challenging step for our data analysis.

Zheng and Klein (1995) [10] evaluate the robustness of the CGE under a misspecified copula model. They find that, as long as the strength of dependency between survival and censoring times is estimated well, the CGE is relatively robust towards misspecification of the copula class. Therefore, we will decide on one copula class, namely the Clayton copula, prior to our data analysis. The Clayton copula can model positive and negative dependency and specifies a straightforward and easy to interpret connection between its parameter θ and Kendall's τ [9, chapter 4,5]. Furthermore, the Clayton copula has successfully been used in previous research to model dependency in survival times, for instance showing good results in modeling the time to metamorphosis for salamander larvae [30], or in analyzing adherence to tuberculosis treatment [6]. Lastly, due to the Clayton copula's simple generator function, we were able to implement a completely vector and matrix‐based algorithm version of the permutation test in Algorithm 1 in R, which saved computing time.

While there are approaches of estimating the dependency parameter from the data, these approaches typically lead to estimators with large variances, especially for small sample sizes [31]. However, there is research on how sensitive statistical inference is to wrongly assumed independence of survival and censoring times. For instance, Siannis et al. (2005) [32] provide bounds around the estimated median survival (calculated under the assumption of independent censoring), depending on the strength and type of dependence between survival and censoring times. Although this can give an idea on how much estimates are biased by wrongly assumed independence, it cannot provide a guideline on how to select the optimal $\tau_{assum.}$ for the proposed test. Instead, a sensitivity analysis can be applied by varying the dependence parameter, evaluating model performance and then using the results to draw conclusions on the underlying dependence structure [7]. Emura and Chen (2016) [4], who introduce an extension of univariate Cox regression for dependent censoring, recommend selecting τ using a cross‐validated Harrell's C‐index. They build their model for various assumed τ, estimate Harrell's C for each model and then choose the model with the largest C‐index. We will use the same approach for our model selection and add the Integrated Brier Score as an additional performance measure. More details can be found in Section 4.1, where the setup of our survival tree study is explained.

## 3 Simulation Study: Tests

### 3.1 Simulation Design

The following simulation study aims to show that the proposed test indeed is a level‐α test with good power and type I error properties. Furthermore, we identify scenarios where the proposed test might fail. To do so, we will simulate data with proportional as well as non‐proportional hazards and perform sensitivity analysis regarding the assumption of equal censoring distributions across groups.

We compare the performance of the permutation test introduced in Section 2.3 using the Clayton copula and assumed concordance parameter $\theta_{assum.}\in \{0.000,0.\bar{6},2,6\}$, which corresponds to $\tau_{assum.}\in \{0.000,0.25,0.5,0.75\}$.^1^ Since logrank test‐based survival trees are commonly found in literature, [15, 18] we additionally included the logrank test. Furthermore, we included the Peto–Peto test that weights the logrank statistic by an estimate of the pooled survival function of both groups, [24, chapter 7] but will only report its results, when they deviate from the traditional logrank test. Both tests were evaluated using the R‐package survival [33].

We set the desired type I error to α=0.05. The type I error, or power, from $n_{sim}$ rounds of simulations is estimated as $\hat{\text{power}}=1/n_{sim}\sum_{i=1}^{n_{sim}}1(p_{perm_{i}}\leq \alpha )$ for $p_{perm_{i}}$ being the p‐value from the ith simulation. The uncertainty of performing a simulation study with a finite number of repetitions is given by the empirical or Monte‐Carlo standard error (SE) as $\sqrt{1/n_{sim}\left(\hat{\text{power}}\times (1-\hat{\text{power}})\right)}$ [34]. Thus, for an estimated type I error of 0.05 from $n_{sim}=2000$ repetitions, the Monte‐Carlo standard error estimate would be 0.005. During power estimation its upper bound is given by $0.5/\sqrt{n_{sim}}$ [35], which is 0.011 for $n_{sim}=2000$ and 0.016 for $n_{sim}=1000$.

The additional insecurity of calculating a permutation test on $n_{perm}$ rather than all n! permutations can approximately be quantified by a factor of 1.2 that is added to the Monte‐Carlo standard error [36]. Both for the type I error and maximal error based on 1000 or 2000 permutations, this seems acceptable for the purpose of getting a general idea of our test's performance. Thus, we choose $n_{sim}=1000$ for calculating power curves and $n_{sim}=2000$ for type I error analysis, where the exact maintenance of the error rate seems relevant. A permutation number of $n_{perm}$ = 1000 is chosen for both cases, which is well above the suggestion of $8\sqrt{n_{sim}}$ by Boos and Zhang (2000) [36].

**FIGURE 1:**
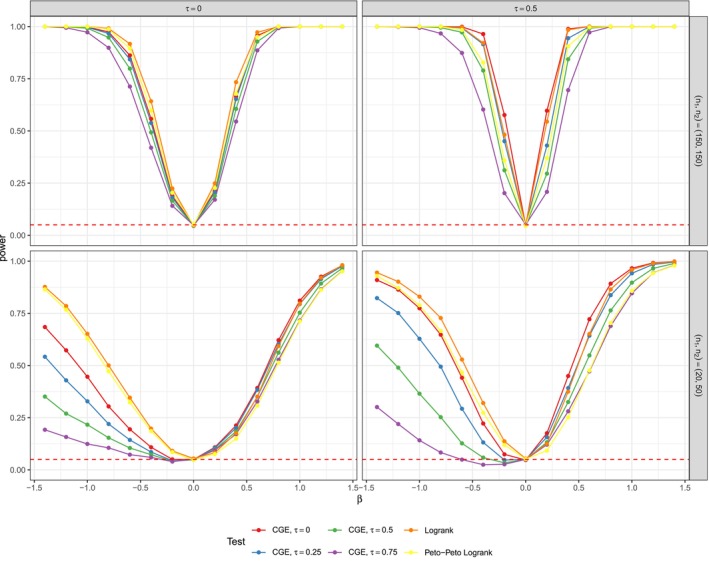
Power estimates with theoretical dependency of event and censoring times of $\tau_{theor.}=0.0001$ (left), and $\tau_{theor.}=0.5$ (right) for $n_{1}=n_{2}=150$ (top) and $n_{1}=20,n_{2}=50$ (bottom). r=0.5 for all Figures. The simulations were performed with $n_{sim}=5000$ and $n_{perm}=5000$. The results are almost identical to simulations with small $n_{sim}=n_{perm}=1000$ in Figure B3.

**FIGURE 2:**
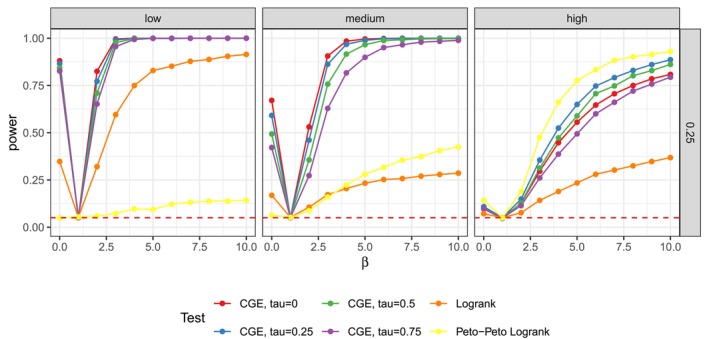
Power estimates for normal covariates with varying standard deviation between groups, $n_{1}=n_{2}=150$ and $\tau_{theor.}=0.25$. Censoring percentages vary across columns from 0.258 and r=0.1 on the left to 0.501 and r=0.5 on the right. The simulations were performed with $n_{sim}=5000$ and $n_{perm}=5000$. The results are almost identical to simulations with small $n_{sim}=n_{perm}=1000$ in Figure B12.

To further confirm our choices of $n_{sim}$ and $n_{perm}$, we re‐ran parts of the type I error and power analysis with larger $n_{sim}=n_{perm}=5000$. The results can be seen in Tables A1, A2 and Figures 1, 2, with the results corresponding to Tables A3, A4 and Figures B3, B12 in the Appendices A and B. The results from the sensitivity analysis regarding type I errors show only minor deviations from the main study. While individual estimates differ slightly from the main study (as expected due to Monte Carlo variability), we observe no systematic deviation that would indicate an over‐ or underestimation of the type I error with smaller simulations. The same holds true for the power analysis.

For type I error analysis, we consider all group sample sizes in $\left(n_{1},n_{2})\in \{20,50,100,200\}\times \{20,50,100,200\right\}$, to cover small to moderately large sample sizes as well as both balanced and unbalanced group sizes. For the computationally more extensive power analysis, which evaluates a larger grid of effect sizes and censoring/data‐generation settings, we consider sample sizes in $\left(n_{1},n_{2})\in \{20,50,150\}\times \{20,50,150\right\}$ ({20,50,150} for the non‐proportional hazards setting), again including balanced and unbalanced settings. These sample size choices are motivated by our PBC study in Section 5, which includes a total of 312 patients that we repeatedly want to split into two groups. Finally, the selected sample sizes are consistent with sample sizes used in other simulation studies on two‐sample survival tests [37, 38].

The software R in version 4.2.1 was used for all calculations [39] and visualizations were made using the ggplot2 package [40].

#### 3.1.1 Proportional Hazards

To simulate survival times from a Cox proportional hazard model, [24, chapter 2] we condition survival times on a one‐dimensional observed covariate z and receive $S_{T}(t|z)=\exp\left(-H_{0}(t)\exp(\beta z)\right)$ for a parameter β∈ℝ and cumulative baseline hazard function $H_{0}$. Since $\exp\left(-H_{0}(T)\exp(\beta z)\right)∼\text{Unif}[0,1]$ for survival time T, we solve this term for T, generate uniform random variables U and then simulate our survival times as $T=H_{0}^{-1}\left(-\log(U)\exp(-\beta z)\right)$ [41]. Since previous simulation studies on survival data showed censoring rates to influence results much more than underlying statistical distributions, we choose simple distributions and focus on modeling our test's properties under various censoring rates [37]. To achieve this, we generate survival times using an exponential model with scale parameter λ=1, such that $H_{0}(t)=\lambda t$ and T=−log(U)exp(−βz).

The generation of survival times depending on covariates is inspired by studies assessing properties of classification models [4, 17, 23], and provides simulation settings that can be generalized to datasets as described in Section 2.1. For the major part of the study, we consider binary covariates with $z_{i}=0$ for subjects from group 1 and $z_{i}=1$ for subjects from group 2. Parameter β varies on [−1.4,1.4], with β=0 representing the null hypothesis. Trial simulations indicated that the interval of [−1.4,1.4] is large enough to observe a power close to 1 towards the edge of the considered β‐interval.

Besides the described regression‐inspired designs with survival times depending on some βz, we additionally assess power on alternatives with varying covariate generating mechanisms between groups and β=1 for both groups, to mimic datasets like the one we will study in Section 5 with survival times possibly depending on various clinical covariates. Namely, we consider:

1. Normally distributed covariates with mean μ=0 in group 1, μ=γ in group 2 and standard deviation σ=1 in both groups. Parameter γ is varied on [−1.5,1.5] with γ=0 describing the null hypothesis.
2. Normally distributed covariates with variance σ=1 in group 1, σγ in group 2 and mean μ=0 in both groups. Parameter γ is varied on [0.00001,10] with γ=1 describing the null hypothesis.
3. Poisson distributed covariates with parameter λ=1 in group 1 and parameter λ=1+γ in group 2. Parameter γ is varied on [−0.9,1.5] with γ=0 describing the null hypothesis.

Censoring times are generated by drawing a second set of times as described above, and setting the minimum of event and censoring time as the observed value [42]. The scale parameter is set to λ=(1/(1−r))−r, such that for β=0 and independent survival and censoring times, the resulting survival data will have a censoring percentage of 100r % [43]. This leads to censoring time $C=-\log(V)\cdot {\left(\left(1/(1-r)\right)-r\right)}^{-1}$ for uniform V. Three stages of censoring are simulated by choosing r∈{0.1,0.25,0.5}. Hence, censoring times are generated independently from covariates and β, to ensure equal censoring distributions across groups, as requested in Methods Section 2.3 (see Section 3.2.3 for a sensitivity analysis regarding the violation of this assumption).

To model dependent survival and censoring times, U,V∼Unif[0,1] are drawn according to a pre‐specified copula model using the R‐package Copula.surv [44]. All simulation settings are tested under two different censoring‐event dependencies, namely the Frank and the Clayton copula, which span notably different dependence regimes within the Archimedean family. While Clayton copulas allow us to model lower‐tail dependence, Frank copulas have no tail dependence. We used the true theoretical dependency parameters $\tau_{theor.}\in \{0.0001,0.25,0.5,0.75\}$. Furthermore, we run simulations with $\tau_{theor.}=0.95$ to understand the behavior of our test under extreme values of $\tau_{theor.}$.

#### 3.1.2 Non‐Proportional Hazards

To investigate the performance of our test under non‐proportional hazards, we use a Gompertz baseline hazard function $h(t)=\exp(\gamma t+\beta_{1}z+\beta_{2}zt)$ to generate time‐dependent effects, resulting in non‐proportional hazards. This hazard function can be analytically integrated and inverted to generate survival times as: [42]

$$T=\frac{1}{\gamma +\beta_{2}z}\log\left(-\frac{\gamma +\beta_{2}z}{\exp(\beta_{1}z)}\log(U)+1\right).$$

We simulate data for $\beta_{1}\in [-1.4,1.4]$, $\beta_{2}\in \{0,0.6,1.4\}$, γ=1, group sizes $n_{1},n_{2}\in \{20,50,150\}$, censoring parameter r∈{0.1,0.5} and dependency of censoring and survival times $\tau_{theor.}\in \{0.0001,0.5\}$.

### 3.2 Simulation Results

#### 3.2.1 Proportional Hazards

In the following, the results of type I error and power analysis will be shown. Unless stated otherwise, the described results in this subsection refer to the settings with Clayton copula modeled dependence and binary covariates. Numbers will be rounded to three digits after the decimal sign.

Under proportional hazards, all tests maintain the type I error rate of 0.05 relatively well in all settings, with 90% of type I error estimates falling into the interval of [0.043,0.060] and all estimates being within [0.037,0.0685]. The median type I error estimate is 0.051. An overview of the estimated type I error rates for moderate sample sizes ($n_{1}=n_{2}=50$) on data generated by the Clayton copula, which in our case is the correctly specified copula class, can be seen in Table A3 in the Appendix A. While the CGE‐based tests show acceptable type I error estimates in all cases, Table A3 does not display any coherent patterns across varying $\tau_{theor.}$ and censoring scenarios. Similar findings were made for other sample sizes.

The logrank test's type I error does not increase substantially when its assumption of independent survival and censoring times is violated. For small sample sizes ($n_{1}=20$ or $n_{2}=20$) and a high dependency parameter of $\tau_{theor.}=0.75$ all tests, but in particular the logrank test, are slightly too liberal. The logrank test has type I error estimates up to 0.067 (SE of 0.006), which can be seen in Table A4 in the Appendix A. For larger n, this problem vanishes, as Table A5 illustrates.

**FIGURE B2:**
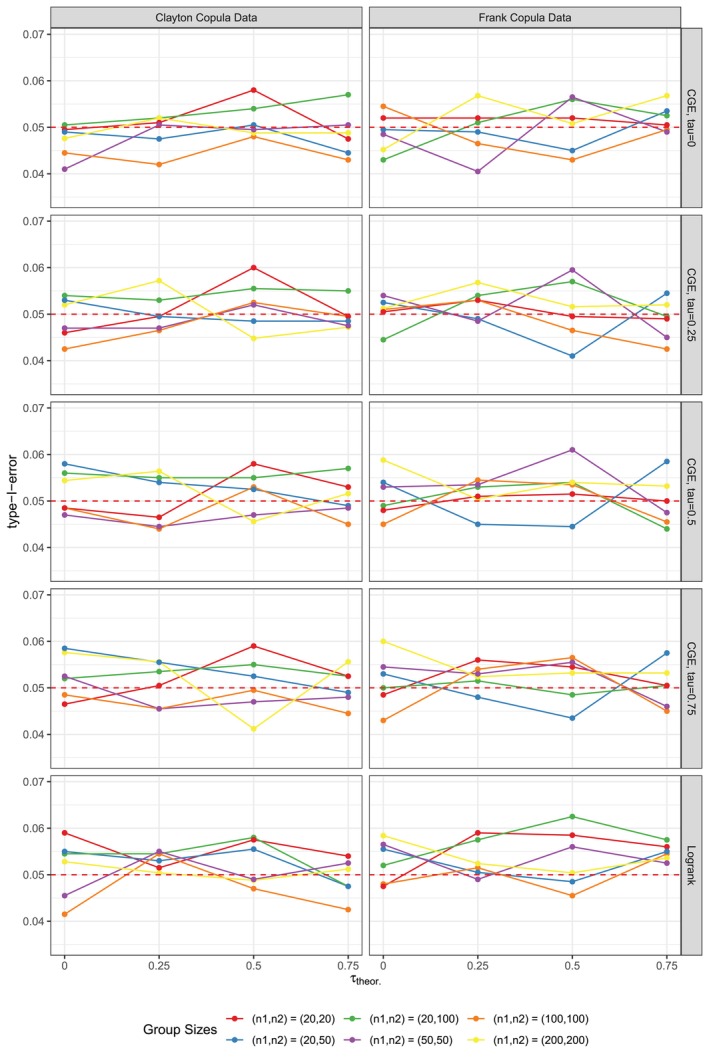
Type I error estimates by copula model, sample size, and test. Each row of the graphic displays the data of one statistical test for r=0.5.

**FIGURE B3:**
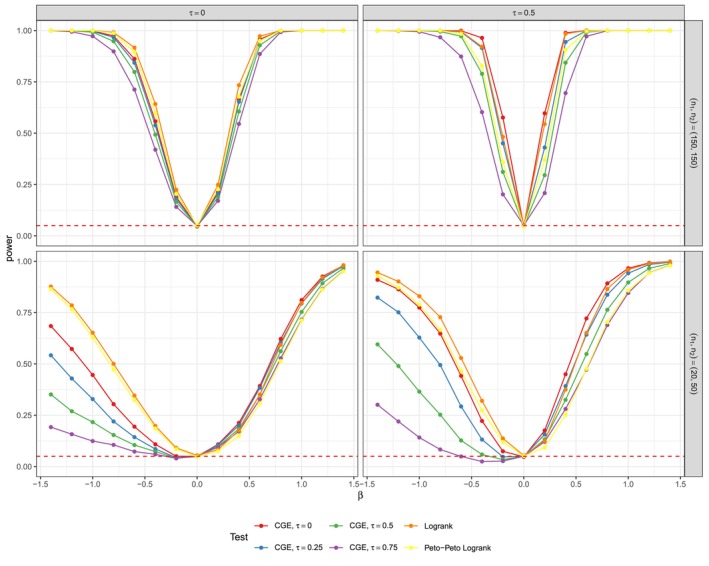
Power estimates with theoretical dependency of event and censoring times $\tau_{theor.}=0.0001$ (left), and $\tau_{theor.}=0.5$ (right) for $n_{1}=n_{2}=150$ (top) and $n_{1}=20,n_{2}=50$ (bottom). r=0.5 for all Figures. Data for this Figure was simulated with $n_{sim}=n_{perm}=1000$. This figure was re‐created as part of the sensitivity analysis with $n_{sim}=n_{perm}=5000$. See Figure 1 for results.

We did not find any systematic difference of type I errors between tests on data generated using a Clayton copula and tests on Frank copula data, which result in a misspecified model. Both copula scenarios had a median type I error of 0.051 over all considered simulation settings, with 90% of the type I errors falling into [0.0435,0.0588] for the Clayton copula and into [0.043,0.060] for the Frank copula, respectively. Figure B2 in the Appendix B gives more insight into this and compares type I error rates across copula models by test, sample size and theoretical dependence $\tau_{theor}$. The data displayed in the graphic is generated with censoring parameter r=0.5, yielding a mean censoring proportion of 0.500. The considered misspecifications of the copula class do not seem to cause inflated or too conservative type I error rates. Furthermore, no trend in type I error rates for varying $\tau_{theor.}$ is visible for data from either copula model. In particular, our study results do not show a superior maintenance of the type I error rates, when the true $\tau_{theor.}$ and the assumed $\tau_{assum.}$ coincide. Again, the logrank test is slightly more liberal than the CGE‐based test. The results for r∈{0.1,0.25} are similar.

Overall, we are satisfied with the type I error of the proposed test and move on to power analysis.

The power estimates for large sample sizes of $n_{1}=n_{2}=150$, binary covariates and simulated dependency of $\tau_{theor.}\in \{0.0001,0.5\}$ can be seen in the upper part of Figure 1. The data for the graphic was generated with high censoring parameter r=0.5. Two things have to be noted: Firstly, the censoring distributions of both groups are identical and independent of β. However, the survival time distributions on the alternative hypothesis vary between groups. Our observations were generated as x=min(t,c) for survival times t and censoring times c. Therefore, the mean censoring percentage of our simulated data in group 1 (which has survival times not affected by β) is constantly around 0.500, but the mean censoring percentage in group 2 varies with β. The deviations in the censoring proportion in both groups range from minor ones (e.g., 0.499 vs. 0.435 for β=0.2 and $\tau_{theor.}=0.5$) to major ones (e.g., 0.501 vs. 0.327 for β=1). More detailed information on censoring percentages of the data from Figure 1 can be found in Table A6. Secondly, the censoring mechanism seems to not be completely independent from $\tau_{theor.}$. Censoring proportions rise with $\tau_{theor.}$ for negative β (e.g., 0.591 for $\tau_{theor.}$ = 0.0001 and 0.646 for $\tau_{theor.}$ = 0.5 for β=−0.6 and r=0.5) and decrease for positive $\tau_{theor.}$ (e.g., 0.425 for $\tau_{theor.}$ = 0.0001 vs. 0.351 for $\tau_{theor.}$ = 0.5 for β=0.6). Thus, while a comparison of the tests within each sup blot in Figure 1 is possible, since the powers were estimated on the same datasets, only a limited interpretation of the performance for varying dependence is reasonable.

All in all, the tests were able to detect deviations from the null hypothesis β=0 and for large |β|, their power estimates are close to 1. In all cases, the CGE tests with lower $\tau_{assum.}$ performed better with the $\tau_{assum.}=0$‐test having the best power. The logrank and Peto–Peto tests perform similarly.

Exemplary, for $\tau_{theor.}=0.5$ and β=−0.4, the CGE‐based test with $\tau_{assum.}=0$ has an estimated power of 0.960 (SE of 0.001). The test for $\tau_{assum.}=0.5$ has a power of 0.783 (SE of 0.013) and the test with $\tau_{assum.}=0.75$ is at 0.587 (SE of 0.016).

**FIGURE B4:**
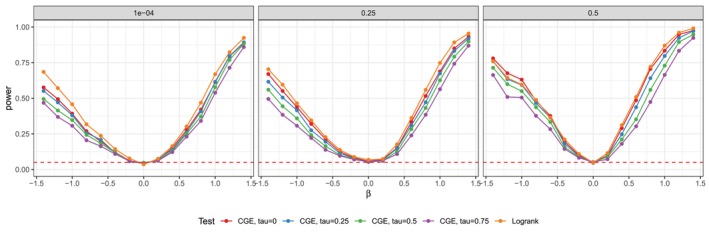
Power estimates with theoretical dependency of event an censoring times of $n_{1}=n_{2}=20$, r=0.5, $\tau_{theor.}=0.0001$ (left), $\tau_{theor.}=0.25$ (middle) and $\tau_{theor.}=0.5$ (right).

All tests showed a faster increase with positive β than with negative β, which partially could be attributed to the varying mean censoring percentages described in Table A6. In both displayed scenarios with $n_{1}=n_{2}=150$ of Figure 1, the logrank test and the CGE‐based test for $\tau_{assum.}=0$ perform similarly. While for $\tau_{theor.}\in \{0.0001,0.25\}$, the logrank test has higher power, the CGE test has a higher power for $\tau_{theor.}=0.5$. For simulation settings with balanced, but smaller group sizes, the power rises slower than for the case of $n_{1}=n_{2}=150$, but the performance of the test relative to each other remains the same. See Figures B4 and B7 in the Appendix B for details.

**FIGURE B5:**
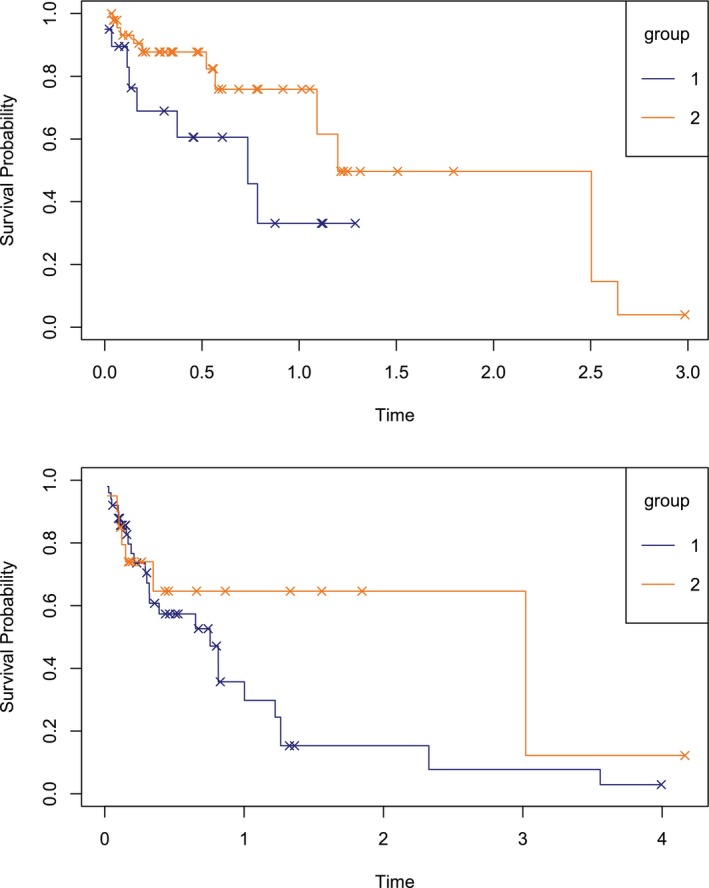
Copula‐graphic estimators with τ=0.25 and the Clayton copula for exemplary data simulated with β=−0.6, $\tau_{theor.}=0.25$ in the Clayton copula, r=0.5 and binary covariates. $n_{1}=20$ and $n_{2}=50$ (top) and $n_{1}=50$ and $n_{2}=20$ (bottom). p‐values top: 0.170 (CGE test with $\tau_{theor.}=0.25$) and 0.031 (logrank test). p‐values bottom: 0.030 (CGE test with $\tau_{theor.}=0.25$) and 0.141 (logrank test).

**FIGURE B6:**
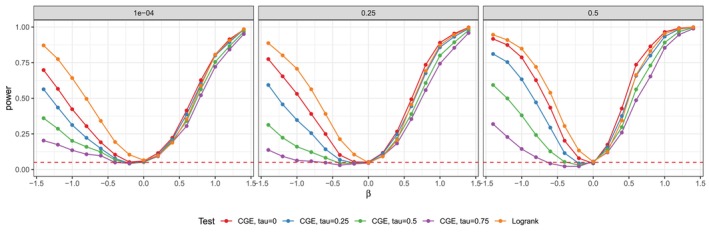
Power estimates for unbalanced sample sizes by $\tau_{theor.}$ for $n_{1}=20$, $n_{2}=50$ and r=0.5.

**FIGURE B7:**
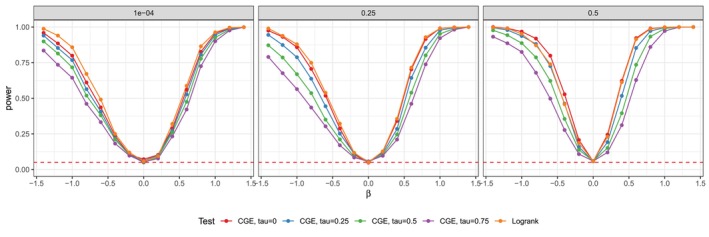
Power estimates with theoretical dependency of event and censoring times of $n_{1}=n_{2}=50$, r=0.5, $\tau_{theor.}=0.0001$ (left), $\tau_{theor.}=0.25$ (middle) and $\tau_{theor.}=0.5$ (right).

**FIGURE B8:**
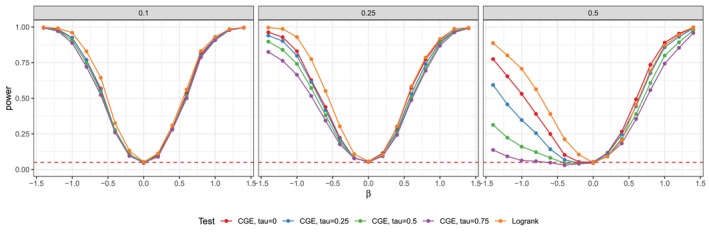
Power estimates for unbalanced sample sizes by censoring parameter r for $n_{1}=20$, $n_{2}=50$ and $\tau_{theor.}=0.25$.

The lower part of Figure 1 illustrates deficits of the CGE tests in unbalanced, small sample size settings. In the case of $n_{1}=20$ and $n_{2}=50$, the logrank test's power rises steadily with β<0 increasing in absolute value. However, the CGE‐based test's power, especially for large $\tau_{assum.}$ increases much slower and even falls to 0.030 (SE of 0.005) at β=−0.4 for the test with $\tau_{assum.}=0.75$. For β<0, group 2 is expected to have longer survival times. This leads to scenarios, where the latest observed time in smaller group 1, $\max(x_{1})$, is a lot lower than the latest time $\max(x_{2})$ in group 2. An example of a single dataset from our simulation study illustrates this in Figure B5 in the Appendix B. The logrank test is able to detect the longer survival of group 2 with a p‐value of 0.031. However, the CGE‐based statistic, only comparing survival curves up to $\min(\max(x_{1}),\max(x_{2}))$, misses out on the fact that there is a large difference in $\max(x_{1})$ and $\max(x_{2})$ and yields a p‐value of 0.170. Apparently, for this setting, the division of the absolute distance in between the CGEs by observation time span $\min(\max(x_{1}),\max(x_{2}))$ is not enough to counteract this. The issue occurred for various values of $\tau_{theor.}$ (see Figure B6), but was less present for lower censoring parameters r (see Figure B8).

The problem vanishes when group 1 has a larger sample size compared to group two, for example, for $n_{1}=50$ and $n_{2}=20$. The larger sample size in group 1 with expected shorter survival leads to smaller differences of $\max(x_{1})$ and $\max(x_{2})$ and thus a larger proportion of the study time is accounted for in the CGE‐based test statistic. In some settings, the CGE tests outperform the logrank test, such as the case of β=−0.2, where the logrank test has a power of 0.057 (SE of 0.007) and the four CGE‐based tests have powers in between 0.089 (SE of 0.009) and 0.098 (SE of 0.009). Again, an illustration of an exemplary dataset is shown in Figure B5.

**FIGURE B9:**
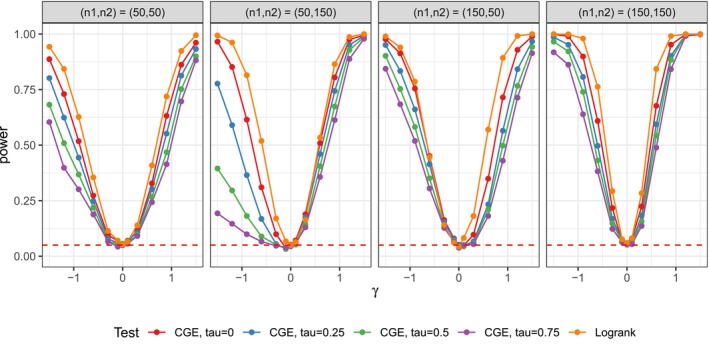
Power estimates for normal covariates with varying mean between groups (see setting **1** in Section 3.1.1). $\tau_{theor.}=0.25$ and r=0.5.

In the following paragraphs, we will discuss results from the three settings with alternative covariate structures. In setting **1.** additional variance is added to simulated times by adding normal covariates of varying means to group 2. Still, the means of the covariates generating the survival times are varied across the same range as β was for binary covariates. Hence, the results of setting **1.** are very similar to the results described above. All tests increase in power for large sample sizes in both groups. Again, the logrank test outperforms the CGE‐based tests in almost all settings. For unbalanced designs with lower $n_{1}$ and smaller covariates in group 1 (i.e., γ<0), the same problems described for binary covariates with β<0 occur and the CGE tests perform worse than the logrank test. The problems vanish if both groups have a smaller sample size. These power properties are illustrated in Figure B9 for the case of $\tau_{theor.}=0.25$ and r=0.5.

**FIGURE B10:**
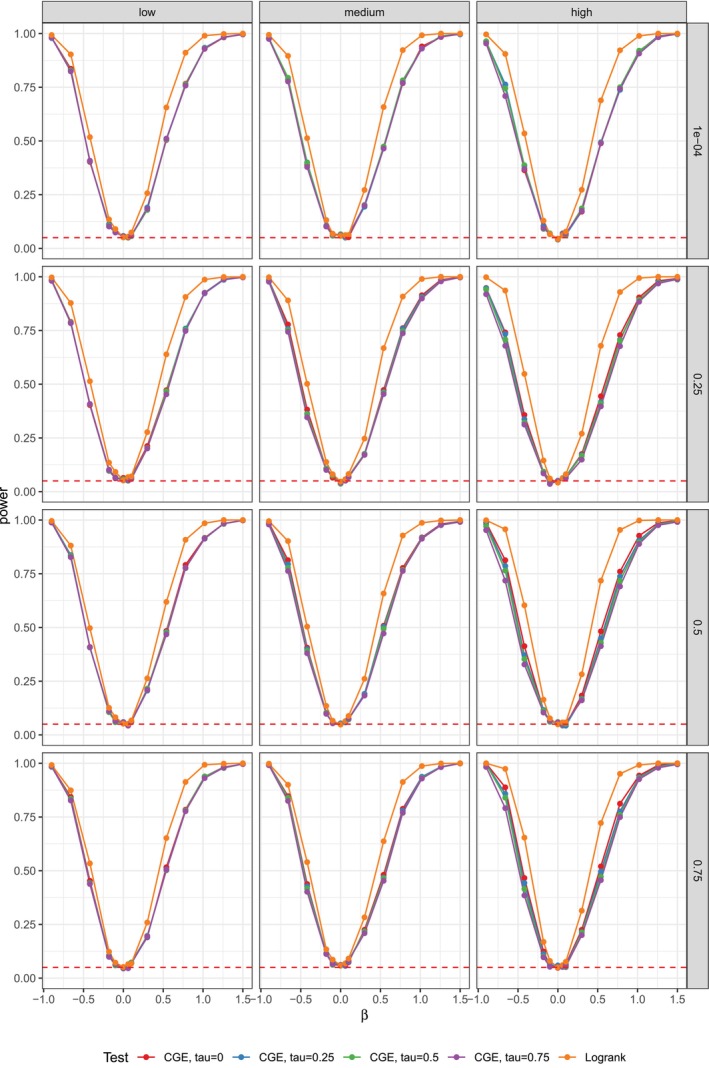
Power estimates for Poisson covariates with $n_{1}=n_{2}=150$ by censoring scenarios r=0.1 (low), r=0.25 (medium) and r=0.5 (high). $\tau_{theor.}$ varies across rows.

Setting **3.** has Poisson distributed covariates with parameter λ=1+γ and γ being varied on [−0.9,1.5] to consider various alternative hypotheses. Thus, mean and variance of the survival time generating covariates differ across groups. Still, all tests are able to maintain the type I error well and the power rises steadily with |γ| and achieves values close to one for large |γ| for all tests. For sample sizes of $n_{1}=n_{2}=150$, this is illustrated in Figure B10. In all settings, the logrank test's power is higher than that of CGE‐based tests, with power curves being clearly separated for all γ. For large sample sizes, the CGE‐based tests have an almost identical performance for all considered $\tau_{assum.}$.

**FIGURE B11:**
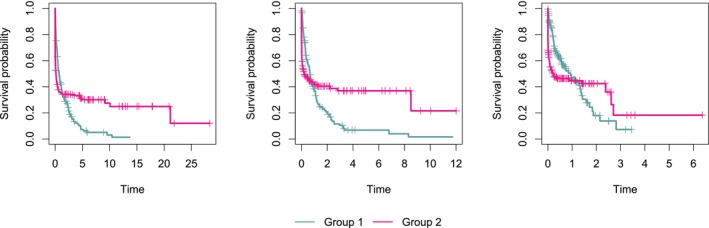
CGEs of both groups for an exemplary dataset from Figure 2 at β=7.5 for low censoring (left), medium censoring (middle) and high censoring (right).

**FIGURE B12:**
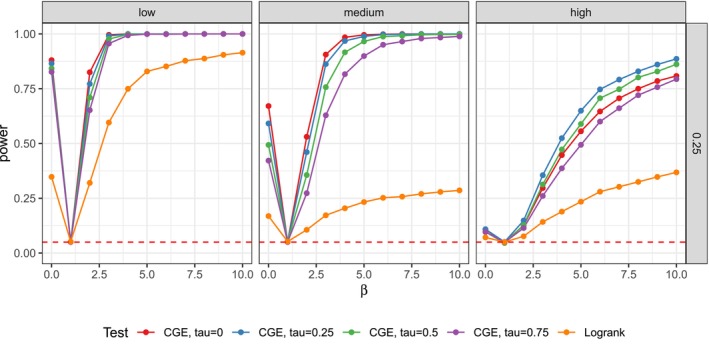
Power estimates for normal covariates with varying standard deviation between groups, $n_{1}=n_{2}=150$ and $\tau_{theor.}=0.25$. Censoring percentages vary across columns from 0.258 and r=0.1 on the left to 0.501 and r=0.5 on the right. Data for this Figure was simulated with $n_{sim}=n_{perm}=1000$. This figure was re‐created as part of the sensitivity analysis with $n_{sim}=n_{perm}=5000$. See Figure 2 for results.

Lastly, setting **2.** has normal covariates and alternative hypotheses of standard deviation 1 in group 1 and varying deviation γ in group 2. The mean of the covariates is the same for both groups. CGE‐based tests generally had higher power, since the logrank test was only able to achieve acceptable power for large n and low censoring rates. For $n_{1}=n_{2}=150$, dependency $\tau_{theor.}=0.25$, γ=10 and a censoring proportion of 0.257 (r=0.1), all four CGE tests have a perfect power estimate of 1.000 (SE of 0.000). For a higher mean censoring proportion of 0.501, the CGE‐based tests lose power and for example, have power estimates between 0.789 (SE of 0.013) and 0.892 (SE of 0.010) at $\tau_{theor.}=0.25$ and γ=10. The logrank test however only has a power estimate of 0.912 (SE of 0.009) and 0.382 (SE of 0.015) in these two settings. This setting is one of the few, where the behavior of the Peto–Peto test strongly differs from the logrank test's. Specifically, the test performs poorly when the censoring percentage is low, but achieves the best performance among all tests when censoring is high. To illustrate this pattern, Figure B11 shows an exemple dataset for each of the three censoring settings at β=7.5. From the displayed CGEs of the two study groups, we see that the difference in observed times between the two groups early on in the study rises with censoring parameter r. Because the Peto–Peto test places greater weight on early survival differences when computing its statistic, this explains its strong performance under high censoring and poorer performance under low censoring.

**FIGURE B13:**
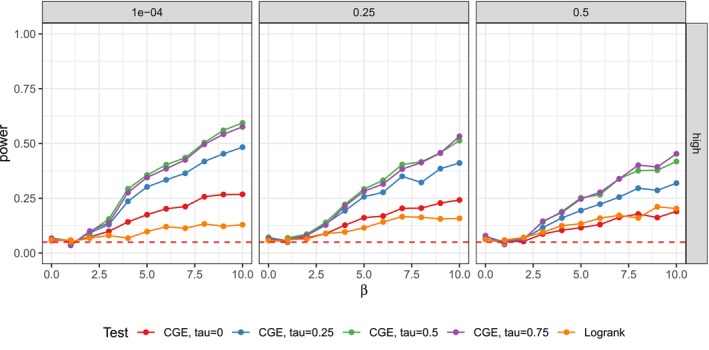
Power estimates for normal covariates with varying standard deviation between groups, $n_{1}=n_{2}=50$ and r=0.5. $\tau_{theor.}$ varies across columns.

Setting **2.** is the only setting where CGE‐based tests with higher $\tau_{assum.}$ repeatedly show greater power than the CGE test with $\tau_{theor.}=0$, as can be seen in Figure 2. In the case of $n_{1}=n_{2}=50$, $\tau_{theor.}=0.5$ and r=0.5 the CGE‐based test with $\tau_{theor.}=0.75$ even has the highest power estimates, which we don't see for any other covariate distributions. See Figure B13 for details. Looking at exemplary datasets from this settings, such as the one displayed in Figure B20, we see that the longest observed times in both groups are similar for large γ. However, in group 2, survivals and censorings are completely separated, meaning that all observed survival times are lower than any observed censoring time. Consequently, the curve of the CGE is at a relatively high level, since the censoring times do not affect the estimator's path at all. The CGE of group 1 on the other hand, especially with large $\tau_{assum.}$, strongly weights censorings in group 1 and falls to a much lower value. Thus, there is a good separation between the estimators of the groups, resulting in a small p‐value. It should be noted that the dataset from Figure B20 was an extreme example regarding the separation of survival and censoring times in group 2, which was not the case for all rounds of simulation.

**FIGURE B14:**
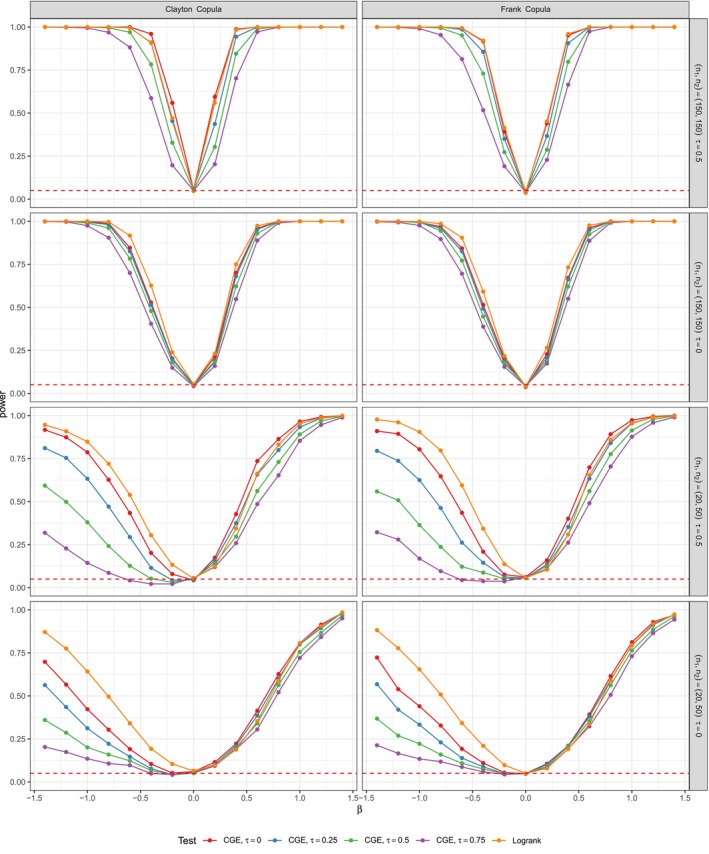
Power estimates with theoretical dependency of event and censoring times of $\tau_{theor.}\in \{0.0001,0.5\}$ and r=0.5. The data in the left column uses a Clayton copula to generate dependencies between survival and censoring times, while the right column uses a Frank copula. All CGE‐based tests are computed using the Clayton copula.

**FIGURE B15:**
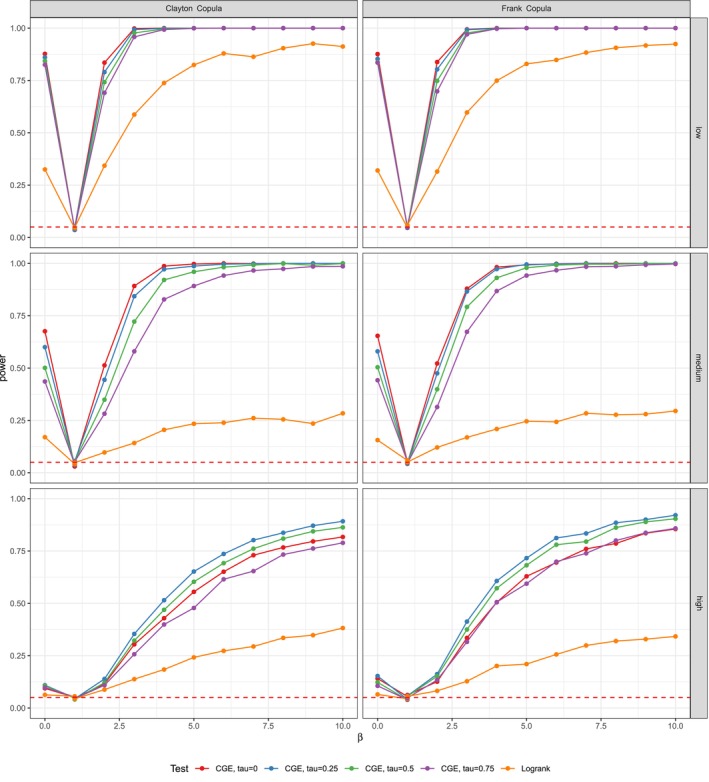
Power estimates for normal covariates with varying standard deviation between groups, $n_{1}=n_{2}=150$ and $\tau_{theor.}=0.25$. We use the censoring parameters of r=0.1 (top), r=0.25 (middle) and r=0.5 (bottom). The data in the left column uses a Clayton copula to generate dependencies between survival and censoring times, while the right column uses a Frank copula. All CGE‐based tests are computed using the Clayton copula.

Similar to our findings on type I error rates, misspecifying the copula class does not seem to reduce the test's power. To illustrate this, we exemplarily study the settings from Figures 1 and 2, and assess how the Clayton Copula–based test's power changes, if we misspecify the copula class. On the left of Figures B14 and B15, dependence between survival and censoring times is generated using a Clayton copula; on the right, we use a Frank copula, which leads to a misspecification of the copula class.

The resulting power curves of the Clayton‐CGE test are very similar, and we do not observe a systematic loss of power when the data are generated from the misspecified (Frank) copula. Small differences occur in individual configurations: For instance the CGE test with $\tau_{assum.}=0.75$ has a power of 0.653 for the Clayton‐based data and a power of 0.704 for the Frank copula generated data (setting of binary covariates, $n_{1}=20$, $n_{2}=50$, β=0.8, censoring parameter r=0.5 and $\tau_{theor.}=0.5$). However, this could also be due to a relatively small number of simulations per setting of $n_{sim}=1000$ or slightly varying censoring percentages across copula models (in this case 26.590% for the Clayton and 27.273% for the Frank copula).

#### 3.2.2 Non‐Proportional Hazards

**FIGURE 3:**
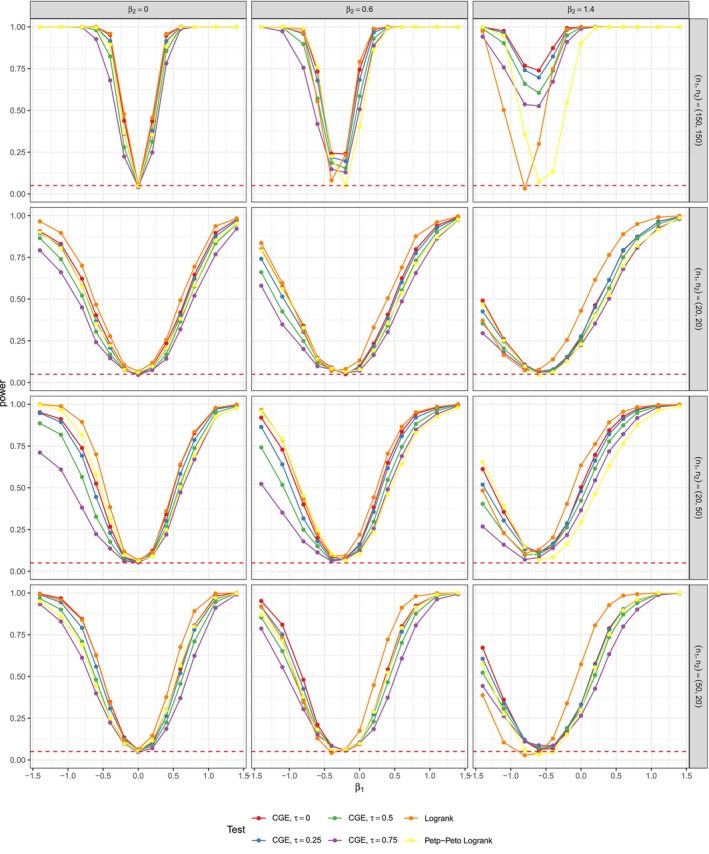
Power estimates with theoretical dependency of event and censoring times of $\tau_{theor.}=0.5$ and censoring parameter r=0.5. The data was generated with non‐proportional hazards.

For the data generated under non‐proportional hazards, we additionally report the results of the Peto–Peto test, since they sometimes differ from those of the logrank test. Under non‐proportional hazards with $\beta_{2}=0$ and a low censoring proportion generated with r=0.1, all tests perform well and very similarly to each other, as can be seen in Figure B17 in the Appendix B. For higher censoring with r=0.5, the tests still have a type I error of about 0.05 and a steadily increasing power with $\beta_{1}$, but the tests' power differs slightly, as seen in Figure 3. The logrank test overall has the best performance and the performance of the CGE tests decreases as $\tau_{assum.}$ increases.

While all tests generally exhibit reasonably high power when $\left|\beta_{1}\right|$ differs considerably from zero, deviations from $H_{0}$ due to $\beta_{2}>0$ are not always detected. For example, consider the setting with $\beta_{1}=-0.8$ and $\beta_{2}=1.4$. Under r=0.5 and $\tau_{theor.}=0.5$, the CGE tests for small sample sizes ($n_{1}=n_{2}=20$) have very low power between 0.085 and 0.107 (see Figure 3, column 3, row 2). Only for the largest sample sizes considered ($n_{1}=n_{2}=150$, column 3 row 1) are the CGE tests able to detect deviations from $H_{0}$, with estimated power ranging from 0.536 (for $\tau_{assum.}=0.75$) to 0.768 (for $\tau_{assum.}=0$). In contrast, the logrank and Peto–Peto tests perform poorly across all sample sizes, with a power of 0.073 for $n_{1}=n_{2}=20$ and only 0.032 for $n_{1}=n_{2}=150$.

These issues can be explained by the way the survival times are generated. We can denote the times as a function of U∈Unif[0,1]:

$$T(U,\gamma ,\beta_{1},\beta_{2},z)=\frac{1}{\gamma +\beta_{2}z}\log\left(-\frac{\gamma +\beta_{2}z}{\exp(\beta_{1}z)}\log(U)+1\right).$$

**FIGURE B16:**
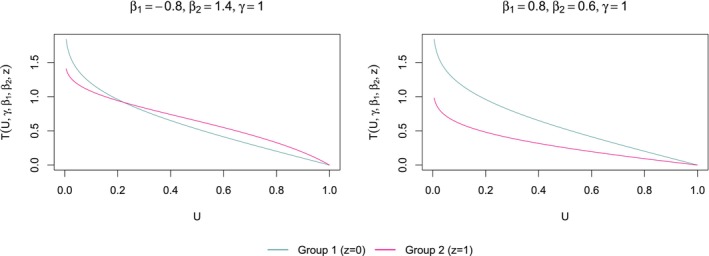
Survival times from two groups generated dependent on uniform variable U∈[0,1] for two different parameter combinations of $\beta_{1}$ and $\beta_{2}$.

Now, there are some parameter settings, where $T(u,\gamma ,\beta_{1},\beta_{2},0)$ (group 1 due to z=0) and $T(u,\gamma ,\beta_{1},\beta_{2},1)$ (group 2 with z=1) are very similar, even though the parameter values of $\beta_{1}$ and $\beta_{2}$ differ from 0. This results in very similar survival times being generated for both groups, even though $H_{1}$ is true. Figure B16 illustrates this for the above mentioned parameter values of $\beta_{1}=-0.8,\beta_{2}=1.4$ and $\beta_{1}=0.8,\beta_{2}=0.6$, where in contrast the generated survival times for the two groups are clearly separated.

**FIGURE B17:**
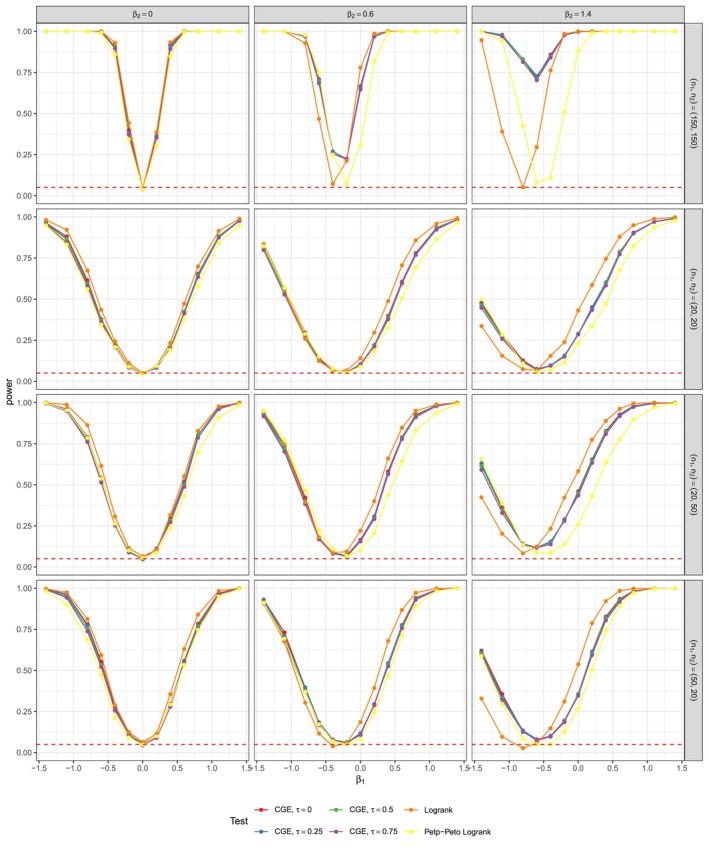
Power estimates with theoretical dependency of event and censoring times of $\tau_{theor.}=0.5$ and censoring parameter r=0.1. The data was generated with non‐proportional hazards.

As Figures 3 and B17 show, the type I error, which should be 0.05, is generally well maintained. Some deviations from 0.05 exist for all tests, including the logrank and Peto–Peto tests. Since they show no clear pattern across $\tau_{theor.}$ and censoring parameter r, they might at least partially be caused by the small number of simulated datasets at $n_{\text{sim}}=1000$. More details for the setting of $n_{1}=n_{2}=150$ can be seen in Table A7 in the Appendix A.

In conclusion, we see that our test is able to provide an accurate type I error to control all considered non‐proportional hazard settings, while it only provides a high power in some of the considered settings. However, the CGE test's power in the setting where it was not ideal, was still comparable and oftentimes better than that of the traditional testing procedures.

#### 3.2.3 Sensitivity Analysis: Censoring Distributions

During the simulations, we observed differences in censoring proportions between the two groups. Although the censoring distributions were identical across groups, group‐specific survival time distributions under $H_{1}$ resulted in different proportions of censored observations. These issues were especially prominent for large $\tau_{theor.}$ and β, which we further investigated for extreme parameter settings $\tau_{theor.}=0.95$ and β∈{−5,5}. Furthermore, we performed a sensitivity analysis to see how our test performs when the assumption of equal censoring distributions for both groups made in Section 2.3 is violated. All Figures shown in this subsection display the mean censoring proportion in both groups in addition to the test's power.

For the sensitivity analysis, we again simulated data with proportional and non‐proportional hazards. We considered violations from the assumption of equal censoring distributions in two ways:

**1: Group‐specific censoring parameters (** r **varies by group):** Censoring times were generated as described in Section 3.2.1, but r differs for both groups with r∈{0.1,0.5} for group 1 and r∈{0.25} for group 2.

**2: Censoring distribution matches survival distribution:** Censoring times are generated the same way that survival times are generated. Consequently, under $H_{1}$ the censoring distributions of the two groups differ.

**FIGURE 4:**
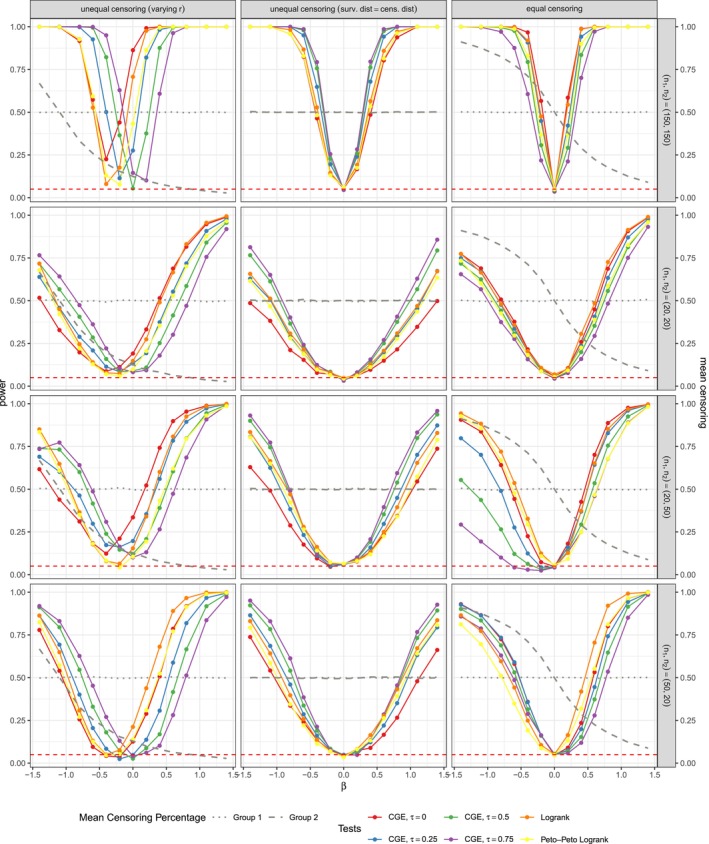
Power estimates with theoretical dependency of event and censoring times of $\tau_{theor.}=0.5$ and proportional hazards. The data was generated with r=0.5 in group 1 and r=0.25 in group 2 (left column), with identical censoring and survival distribution (middle column), and with equal censoring distribution with r=0.5 (right column).

Results for the proportional hazards setting with $\tau_{theor.}=0.5$ are shown in Figure 4. In the setting with equal censoring distributions (left column), censoring times in both groups were generated with r=0.5. For the setting of varying r across groups, censoring in group 2 was generated with r=0.25.

When the censoring distribution equals the survival distribution (middle column), all tests across all sample sizes show a good type‐I error and a steadily increasing power with |β|. In some cases, the test's power is lower compared to the setting with equal censoring distribution, but this could be caused by a higher mean censoring rate in group 2. Overall, our test appears not to be too sensitive to this particular violation of the equal censoring assumption.

However, when censoring distributions differ due to varying values of r, all tests, including the logrank test, perform worse, especially with respect to the type I error control. For the largest sample size considered ($n_{1}=n_{2}=150$), only the CGE test with $\tau_{assum.}=0.5$ maintains an accurate type I error of 0.054. In contrast, the CGE test with $\tau_{assum.}=0$ exhibits the largest type I error (0.863), while the logrank test also shows a substantial type I error inflation of 0.707. Notably, although censoring proportions are lower in this setting than in the case of equal censoring, the tests still perform worse. This indicates that the tests are highly sensitive towards this violation of the assumption of equal censoring distributions.

**FIGURE B18:**
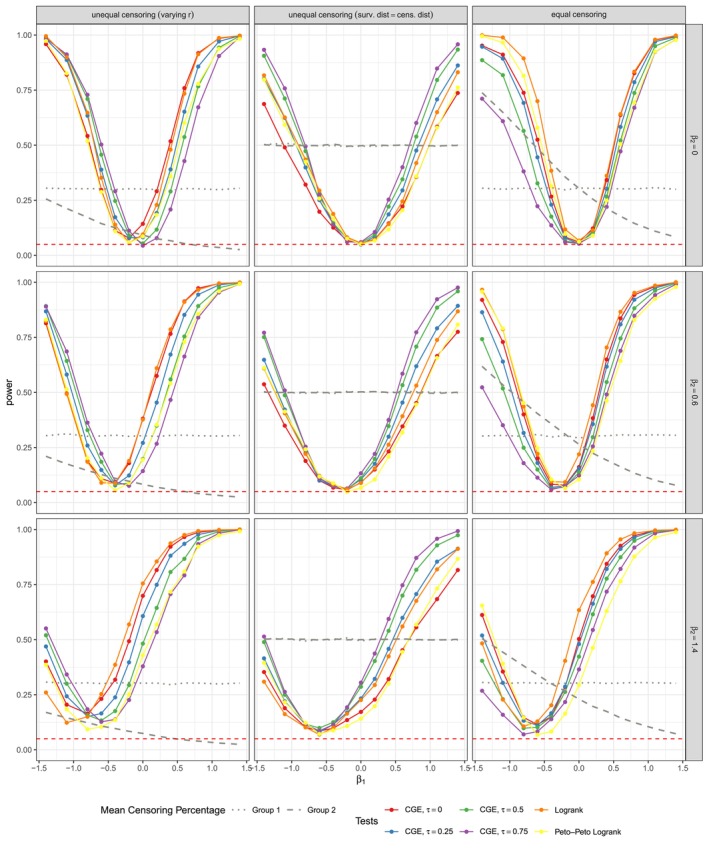
Power estimates with theoretical dependency of event and censoring times of $\tau_{theor.}=0.5$, $n_{1}=20$, $n_{2}=50$ and non‐proportional hazards. The data was generated with r=0.5 in group 1 and r=0.25 in group 2 (left column), with identical censoring and survival distribution (middle column), and with equal censoring distribution with r=0.5 (right column).

For survival times generated under non‐proportional hazards, this issue is less prominent, as can be seen in Figure B18 in the Appendix B. For $n_{1}=20$, $n_{2}=50$, $\tau_{theor.}=0.5$, and the same values of r as above, the type I error under censoring with varying r across groups ranges from 0.044 for the CGE test with $\tau_{assum.}=0.75$ to 0.143 for the CGE test with $\tau_{assum.}=0$. As before, we observe that the power of the tests remains low when $\beta_{2}\neq 0$, which is consistent with the behavior already seen in Section 3.2.2, where the assumption of equal censoring distributions was not violated.

Overall, the sensitivity of our test to violations of the equal censoring distribution seems to depend on the exact form of the deviation. Some violations led to inflated type I error. However, in these settings the traditional logrank and Peto–Peto tests also failed to control the type I error.

To assess the effects of extreme values of $\tau_{theor.}$, we simulated data with $\tau_{theor.}=0.95$, again considering equal as well as unequal censoring distributions between groups. While the mean censoring proportion in group 1 remained constant at 0.5 for uniform censoring times, the censoring proportion in group 2 varies substantially, ranging from nearly 100% for β<0 to almost 0% for β>0. In the setting with equal censoring distributions across groups, this large difference in mean censoring proportions apparently helps all tests to detect differences between the groups and the power improves compared to the setting with $\tau_{theor.}=0.5$.

**FIGURE B19:**
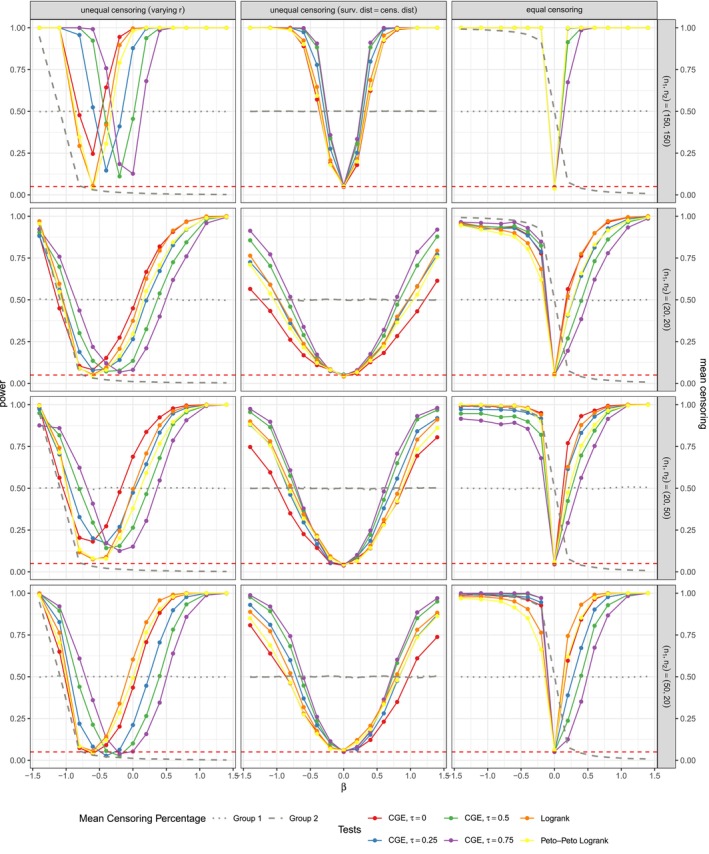
Power estimates with theoretical dependency of event and censoring times of $\tau_{theor.}=0.95$ and proportional hazards. The data was generated with r=0.5 in group 1 and r=0.25 in group 2 (left column), with identical censoring and survival distribution (middle column), and with equal censoring distribution with r=0.5 (right column).

**FIGURE B20:**
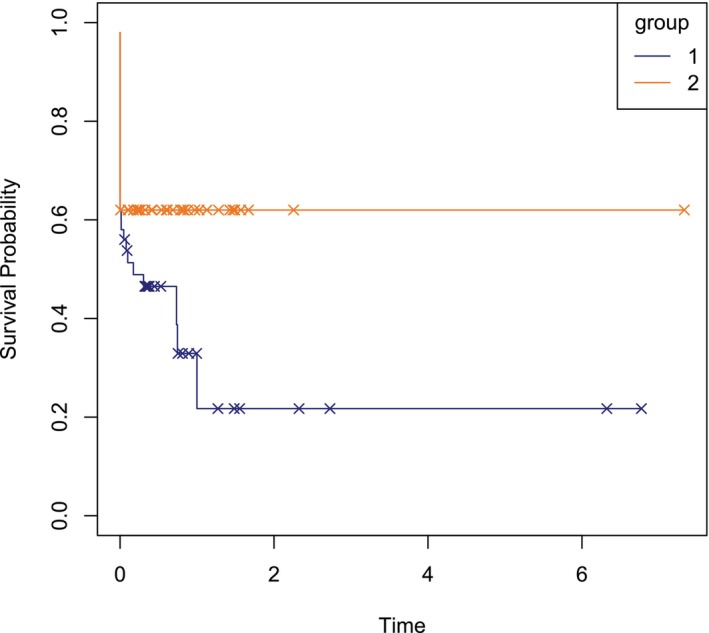
Copula‐graphic estimators with τ=0.25 and the Clayton copula for exemplary data simulated with normal covariates with mean 0, standard deviation 1 in group 1 and standard deviation γ=10 in group 2. $\tau_{theor.}=0.5$ in the Clayton copula, r=0.25 and $n_{1}=n_{2}=50$. p‐values: 0.032 (CGE test with $\tau_{theor.}=0.5$) and 0.236 (logrank test).

For the setting with unequal censoring distributions due to varying r, the tests also show acceptable power for β≠0, which slightly improves with increasing $\tau_{theor.}$. However, the issues with inflated type I error rates discussed above persist. Overall, the CGE tests appear robust to extreme values of $\tau_{theor.}$, even though these induce large differences in mean censoring proportions under our model assumptions. The full results can be seen in Figure B19 in the Appendix B.

To assess the influence of extreme values of β, we simulated data with β∈{−5,5} for the proportional hazards model for all parameter settings (with respect to $n,r,\tau_{theor.}$ and censoring distribution) discussed in Section 3. This led to extreme censoring proportions for data generated under the assumption of equal censoring distributions, with censoring in group 2 close to 100% for β=−5 and close to 0% for β=5. Nevertheless, all CGE tests achieved a power close to 1 across the considered settings, as reflected by the summary statistics for the estimated power shown below (Table 1):

**TABLE 1:** Power under β∈{−5,5} of all considered CGE‐based tests.

Min.	1st Qu.	Median	Mean	3rd Qu.	Max.
0.9700	1.0000	1.0000	0.9997	1.0000	1.0000

Therefore, we assume that our tests are quite robust towards great deviations in censoring proportions under extreme values of β.

## 4 Simulation Study: Trees

### 4.1 Simulation Design

In a second simulation study, we compare the performance of the trees introduced in Section 2.4 to those of the logrank tree regarding prediction ability and ability to select relevant covariates. The study design is motivated by Emura et al. (2012) [45] who generate datasets of survival times and according informative and non‐informative covariates to study methods for compound covariate prediction.

For each round of simulation, we simulate a training and a testing dataset with n=100 subjects each as follows: For subject i, the covariate vector

$$\begin{array}{ll} z_{i} & =(\underset{\times q}{\underbrace{z_{i1},\ldots ,z_{iq}}},\underset{\times q}{\underbrace{z_{i(q+1)},\ldots ,z_{i(2q)}}},\underset{\times p-2q}{\underbrace{z_{i(2q+1)},\ldots ,z_{ip}}}),\;p,q\in \mathbb{N},p>2q, \end{array}$$

is drawn with each entry following a continuous uniform distribution with mean 0 and standard deviation 1. The covariates are separated into three blocks (1 to q, q+1 to 2q and 2q+1 to p). Covariates of the first two blocks have a pairwise correlation of ρ within their block; the remaining genes are uncorrelated. Our data generation mechanism is motivated by *Scenario 2 (gene pathway)* from Emura et al. (2012) [45]. The underlying idea is that covariates can be grouped into blocks representing biological pathways, where each pathway consists of q correlated genes that jointly affect survival in a coherent direction. Restricting pairwise interactions to occur within blocks reflects the common assumption that interactions are more likely among functionally related variables (e.g., genes within the same pathway), and allows us to study the behavior of the tests under a controlled and easy to interpret correlation structure. This block‐structured approach of simulating survival data was used in multiple prior works [4, 45, 46]. We use the X.pathway function from the R‐compound.Cox package to generate datasets of this format [47].

To mimic a clinical dataset consisting of categorical patient data and laboratory biomarker assessments, half of the genes in each block are transformed to a binary scale by calculating ${\text{median}}_{i}(z_{ij})$ for each covariate j∈{1,…,p} and setting the new, binary covariate to ${\tilde{z}}_{ij}=1\{z_{ij}\geq {\text{median}}_{i}(z_{ij})\}$. Subsequently, all covariates are rounded to one digit after the decimal point, which reduces the size of possible cutoff‐values within survival trees and thus greatly speeds up the simulation study. Survival and censoring time are generated analogously to Section 3.1, using a multidimensional parameter

$$\beta =(\underset{\times q}{\underbrace{\beta ,\ldots ,\beta }},\underset{\times q}{\underbrace{-\beta ,\ldots ,-\beta }},\underset{\times p-2q}{\underbrace{0,\ldots ,0}})$$

to generate $T=-\log(U)\exp(-\beta^{⊤}z_{i})$ for uniform U and one‐dimensional auxiliary parameter β. Note that only the first 2q covariates actually affect the survival times. Again, the Clayton copula with parameter $\tau_{theor.}$ is used to generate dependency between survival and censoring times. We compare the performance of CGE‐based trees for $\tau_{assum.}\in \{0.0001,0.25,0.5,0.75\}$ and the logrank tree using the implementation of the uni.survival.tree package [48]. We consider larger sample sizes of n∈{100,300}, in order to have more subject observations than the number of covariates, which was chosen as p=50. As this is only a small simulation study, all other parameter values are not varied, but approximately set in the middle of their respective feasible ranges, which leads to β=0.5, ρ=0.5, p‐value threshold $\tilde{p}=0.01$, and $\tau_{theor.}=0.25$. Two censoring scenarios are considered leading to mean censoring proportion of 0.135 and 0.464, respectively. $n_{sim}=100$ simulation rounds were performed and the number of permutations for the permutation tests was set to $n_{perm}=1000$, which allows us to conduct the study in a manageable amount of time. The trees are evaluated regarding three criteria:

1.
Selection ability: We evaluate, how many of the tree's splits are based on the informative covariates 1 to 2q. We provide the precision, which is the proportion of non‐terminal nodes that split depending on one of the informative covariates. It can take values between 0 and 1, with 1 indicating perfect precision [17].
2.
Prognostic ability: We use Harrell's C‐index to measure, if a tree's terminal nodes are able to order the test dataset's survival times correctly into groups from low to high survival. The terminal nodes are numerated from right to left with low numbers indicating low risk. For any two patients, the index describes, if the patient with the lower terminal node number survived longer than the patient with the higher node number [49]. The index is computed by a pairwise comparison over all test subjects, where one certainly had an event before the other one. If ${\left(tn\#\right)}_{i}$ denotes the terminal node number of subject i, with a low number indicating long survival, the numbers of concordant (CC), discordant (DC) and tied (TR) pairs of all comparable subjects i,j=1,…,n are given by: [50]
$$\begin{array}{ll} CC & =\underset{i,j}{\sum }1\{x_{i}>x_{j}\}1\{{\left(tn\#\right)}_{i}<{\left(tn\#\right)}_{j}\},\\ DC & =\underset{i,j}{\sum }1\{x_{i}>x_{j}\}1\{{\left(tn\#\right)}_{i}>{\left(tn\#\right)}_{j}\},\\ TR & =\underset{i,j}{\sum }1\{x_{i}>x_{j}\}1\{{\left(tn\#\right)}_{i}={\left(tn\#\right)}_{j}\}. \end{array}$$
  The resulting index
$$H_{C}=\frac{CC+0.5TR}{CC+DC+TR}$$
  can take values in [0,1], with high values indicating good prognostic ability [50]. The survival package was used to calculate Harrell's C [33].
3.
Prediction ability: The Brier Score is used to evaluate, how accurately a tree predicts the survival of a subject from the testing dataset at a given timepoint. We predict the survival of a subject in terminal node (tn#) at time t as the value of the CGE calculated on subjects in node (tn#) from the training dataset at time t. We calculate the CGE using the same $\tau_{assum.}$ used to construct the respective tree, and use $\tau_{assum.}\approx 0$, that is, the Kaplan–Meier estimator for the logrank tree predictions. Graf et al. (1999) [51] provide a version of the Brier score that incorporates censoring by weighting deviations of predicted survival from true survival with an estimator of the censoring distribution. This score can then be integrated over the study period to yield the Integrated Brier Score (IBS), which is
(7) $$\begin{array}{ll} IB & =\frac{1}{\max(x)}\frac{1}{n}\sum_{i=1}^{n}\left\{\int_{0}^{x_{i}}\frac{{\left(1-\hat{S}(t|z_{i})\right)}^{2}}{\hat{C}(t)}dt\right.+\left.\delta_{i}\int_{x_{i}}^{\max(x)}\frac{\hat{S}{\left(t|z_{i}\right)}^{2}}{\hat{C}(x_{i})}dt\right\}, \end{array}$$
  with Ŝ and Ĉ describing estimators of survival or censoring function, respectively [50]. Common choices are Kaplan‐Meier estimators. We additionally provide the IBS using CGEs with $\tau_{assum.}=\tau_{theor.}$, which in the case of this simulation study is known to us. The integral from Equation (7) is estimated over a grid of timepoints ${\tilde{t}}_{1},\ldots ,{\tilde{t}}_{m}$ as
$$\begin{array}{ll} \hat{IB} & =\frac{1}{\max(x)}\sum_{j=1}^{m-1}\left\{({\tilde{t}}_{j+1}-{\tilde{t}}_{j})\frac{1}{n}\sum_{i=1}^{n}\left\{\frac{{\left(1-\hat{S}({\tilde{t}}_{j}|z_{i})\right)}^{2}}{\hat{C}({\tilde{t}}_{j})}1\{x_{i}\geq \tilde{t_{j}}\}+\frac{\hat{S}{\left({\tilde{t}}_{j}|z_{i}\right)}^{2}}{\hat{C}(x_{i})}1\{x_{i}<\tilde{t_{j}}\}\right\}\right\}. \end{array}$$
  It is sufficient to choose $\tilde{t}$ as the timepoints observed in the testing or training dataset, since the step‐function like estimators will not fluctuate in between these points.^2^

### 4.2 Simulation Results

Table 2 displays the study results. The performance of the four CGE‐based trees is very similar in all settings regarding the number of terminal nodes as well as all accuracy measures introduced in Section 4.1. No noticeable trends related to $\tau_{assum.}$ can be seen.

**TABLE 2:** Average performance measures from 100 rounds of simulation. $H_{C}$ denotes Harrell's C‐index, $\hat{IB}$ KM is the IBS and $\hat{IB}$ CGE the IBS with the CGE with τ=0.25. Mean censoring proportions of 0.135 (low) and 0.464 (high).

			Copula‐graphic estimator				
			$\tau_{assum.}=0$	$\tau_{assum.}=0.25$	$\tau_{assum.}=0.5$	$\tau_{assum.}=0.75$	Logrank
n=100	low cens.	# term. nodes	15.800	**15.700**	15.760	15.920	24.450
		Precision%	0.765	0.762	**0.776**	0.752	0.619
		$H_{C}$	0.715	0.712	0.711	0.715	**0.718**
		$\hat{IB}$ KM	0.127	0.120	0.118	**0.115**	0.241
		$\hat{IB}$ CGE	0.358	**0.336**	0.356	0.354	0.420
	high cens.	# term. nodes	**13.330**	13.480	13.550	13.750	17.660
		Precision%	**0.799**	0.789	0.762	0.778	0.650
		$H_{C}$	0.730	**0.736**	0.732	0.733	0.728
		$\hat{IB}$ KM	**0.286**	0.288	0.296	0.286	0.340
		$\hat{IB}$ CGE	**0.459**	0.492	0.531	0.528	0.526
n=300	low cens.	# term. nodes	46.520	46.410	**46.120**	46.480	65.810
		Precision%	**0.682**	0.681	0.669	0.666	0.529
		$H_{C}$	0.712	0.715	0.712	0.715	**0.732**
		$\hat{IB}$ KM	0.109	0.108	**0.101**	0.107	0.214
		$\hat{IB}$ CGE	0.396	0.420	**0.391**	0.422	0.521
	high cens.	# term. nodes	39.280	38.760	**38.580**	38.810	46.300
		Precision%	**0.710**	0.680	0.681	0.674	0.557
		$H_{C}$	0.740	0.738	0.742	0.744	**0.753**
		$\hat{IB}$ KM	0.289	0.291	**0.283**	0.292	0.355
		$\hat{IB}$ CGE	**0.511**	0.555	0.566	0.576	0.601

The logrank trees have more terminal nodes than the CGE trees. For instance, the CGE tree with $\tau_{assum.}=0.25$ has 15.7 terminal nodes on average for n=100 and low censoring of 0.135%, while the logrank tree has 24.450, which is the 1.557‐fold. As n rises from 100 to 300, the number of terminal nodes increases with a factor of about 3 for the CGE trees (e.g., factor 2.926 for $\tau_{assum.}=0.5$ and low censoring) and a factor around 2.7 for the logrank tree (2.692 for low censoring). These large numbers for n=300, especially seen for the logrank trees, might diminish the easy and practical interpretability of survival trees. Adjusting the threshold p‐value $\tilde{p}$ or adding a minimum nodesize threshold to allow an additional split to the tree algorithm would solve this problem. Lastly, the trees are smaller for higher censoring, which aligns with the results from Section 3.2, where we saw that both logrank and CGE‐based tests lose power with higher censoring and therefore find fewer significant splits.

The precision of the CGE trees is over 0.666 in all settings. Hence, the majority of covariates is selected correctly. The logrank tree's precision is lower in all settings. For all trees, the precision decreases with higher n (e.g., from 0.762 for n=100 to 0.681 for n=300 for $\tau_{assum.}=0.5$), which might be partially caused by a higher number of cutoff values and thus the higher number of possible splits that comes with larger n. In all settings except for n=100 and low censoring, the CGE test with $\tau_{assum.}=0$ has the largest precision ranging between 0.682 and 0.799. However, the differences are small (e.g., 0.710 for $\tau_{assum.}=0$ vs. 0.681 for $\tau_{assum.}=0.5$ for n=300 and high censoring).

Both the traditional IBS and the CGE adjusted score have higher values for higher censoring, indicating less prediction ability. For instance, the score increases from 0.120 for low censoring to 0.288 for high censoring for the CGE test with $\tau_{assum.}=0.25$ and n=100. In most settings, the logrank test shows the highest values for both versions of the IBS.

Between n=100 and n=300 changes in the Kaplan‐Meier estimate based IBS can be seen, and for multiple trees the values increase. For instance, the logrank test's IBS increases from 0.34 for n=100 to 0.355 for n=300 under high censoring, while its C‐index improves. As the size of the trees (measured as the number of terminal nodes) also increases from n=100 to n=300, this indicates a trade‐off between model complexity and predictive accuracy. However, we believe the effect is driven less by the absolute size of the trees per se, and more by the occurrence of terminal nodes with very few observations (i.e., very small terminal nodes), which occurs more frequently in larger trees.

The IBS itself is computed from subject‐specific survival predictions obtained from the terminal node model fitted on the training data, that is Kaplan‐Meier for the logrank tree and CGE‐based prediction for the CGE trees. When terminal nodes contain only very few training observations, these node‐level survival estimates can become unstable, which can inflate IBS for test subjects assigned to such nodes. This phenomenon is particularly pronounced for the larger logrank trees as can be seen in Table 3.

**TABLE 3:** Number of terminal nodes with k subjects for four exemplary survival trees calculated during the simulation study in Section 4 of our paper (low censoring scenario).

		Terminal nodes with k subjects			
		k=1	k=2	k=3	k≥4
CGE tree ($\tau_{assum.}=0.25$)	n=100	0	0	2	16
	n=300	0	0	4	43
Logrank tree	n=100	4	5	0	12
	n=300	10	11	12	31

The CGE‐based IBS showed larger increases from n=100 to n=300, for example, from 0.354 to 0.422 for low censoring and the test with $\tau_{assum.}=0.75$. In addition to the large number of small terminal nodes mentioned above, this could also be caused by the CGE Score putting higher weights on individuals with very high observed times, which more frequently occur in larger datasets. Details can be found in Appendix C.

For Harrell's C‐index, the row‐wise values of all trees are comparable. In particular, the logrank trees have the highest C‐index in three out of four settings (for instance a value of 0.732 for n=300 and low censoring). All C‐indices are well over 0.500, which would indicate trees that order subjects no better than random assignment. The C‐indices are slightly higher in the high censoring scenario, but do not change much with n.

## 5 Illustrative Data Analysis

In this section, we will apply the proposed tree algorithm to real‐world data. We will use the Primary Biliary Cholangitis (PBC) dataset provided by the survival package [33]. PBC is a progressive liver disease causing inflammations in the liver that lead to cirrhosis, destroy the bile ducts and eventually result in death. The dataset is from a randomized Mayo Clinic trial conducted between 1974 and 1984 testing D‐penicillamine, a possible treatment for PBC [53, chapter 3].

The full dataset contains data from 418 subjects. However, 106 did not participate in the randomized trial and consequently have many missing values in variables potentially very relevant to survival, in particular the variable *Treatment*. Thus, these 106 subjects are removed prior to our data analysis, leaving us with a sample size of 312.

In addition to each patient's event or censoring time, 17 covariates are provided. These include seven binary or categorical variables such as *Presence of Ascites* and *Presence of Hepatomegaly or Enlarged Liver*. Furthermore, ten continuous variables, mostly biomarkers, such as *Serum Bilirubin* or *Serum Cholesterol* are provided. Before analysis, we rounded the variable *Age* to full years to reduce the value of feasible cutoff values and save computation time.

1.080% of covariate observations are missing, especially of the variables *Serum Cholesterol* (8.974% missing) and *Triglycerides* (9.615% missing). So far, the proposed tree cannot handle missing values. Consequently, these two variables were removed from the dataset prior to our analysis. After this, only six subjects with missing values in one or more variables were left. These subjects were removed as well, leaving 306 subjects for the following analysis.

Of the remaining patients, 123 died during the study, 164 were censored due to lost follow‐up and 19 had a liver transplantation. For this illustrative data example, these patients are considered censored as well, since the transplantation prevented a death through PBC.

It is reasonable to assume, that a patient's health status, and with it their underlying survival time distribution, affected the decision, if a liver transplantation was necessary [7]. Thus, we suspect that the dataset was generated by positively dependent survival and censoring times, making it a suitable dataset to test our method.

Before constructing a survival tree, we evaluate the performance of the tree construction algorithms by cross‐validation. We evaluate the performance of the CGE trees using the Clayton copula with Kendall's $\tau_{assum.}\in \{0.0001,0.125,0.250,0.375,0.500,0.625,0.750,0.875\}$ and the logrank tree. We use a p‐value threshold of $\tilde{p}$ = 0.01, the number of permutations $n_{perm.}=5000$ and apply the metrics introduced in Section 4.1 to evaluate the tree algorithms. The performance measures are computed using 10‐fold cross‐validation [54, chapter 7].

The results can be seen in Table 4. The logrank tree has 27.3 terminal nodes on average, which is slightly more than the CGE trees, which have between 19.2 terminal nodes for $\tau_{assum.}\in \{0.375,0.875\}$ and 20.9 terminal nodes for $\tau_{assum.}=0.625$. The larger size of the logrank tree was also seen in the simulation study in Section 4.2. The mean C‐index of the CGE‐trees ranges from 0.769 to 0.801, with no clear trend in relation to $\tau_{assum.}$ visible. The logrank tree had the highest C‐index of 0.816.

**TABLE 4:** Mean performance measures on PBC data over 10‐fold cross validation. $H_{C}$ denotes Harrell's C‐index and $\hat{IB}$ KM is the IBS based on the Kaplan‐Meier estimator. The mean censoring proportion is 0.598.

		$\tau_{assum.}$ of CGE							
Tree	Logrank	0.000	0.125	0.250	0.375	0.500	0.625	0.750	0.875
# term. nodes	27.3	19.3	19.9	20.3	**19.2**	19.3	20.9	19.4	**19.2**
$\hat{IB}$ KM	0.192	**0.181**	0.189	0.201	0.194	0.203	0.203	0.211	0.212
$H_{C}$	**0.816**	0.791	0.769	0.782	0.790	0.801	0.796	0.789	0.772

The Kaplan–Meier–based IBS increases almost monotonously with $\tau_{assum}$. Only the trees with $\tau_{assum.}=0$ and $\tau_{assum.}=0.125$ have a lower Score than the logrank tree at 0.192, while the CGE tree with $\tau_{assum.}=0.375$ almost ties the logrank tree at a Score of 0.194.

**FIGURE B21:**
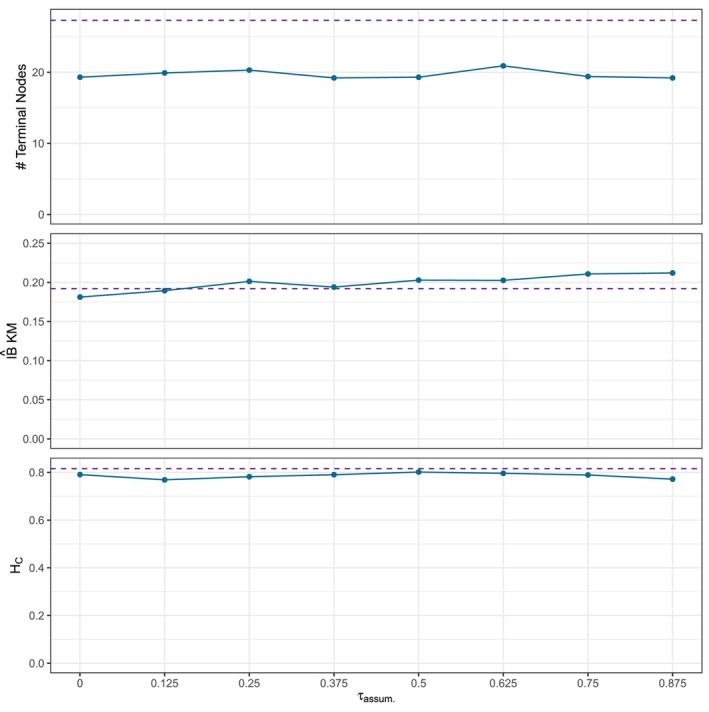
Mean performance measures on PBC data over 10‐fold cross validation. $H_{C}$ denotes Harrell's C‐index and $\hat{IB}$ KM is the IBS based on the Kaplan–Meier estimator. The solid lines display the data of the CGE trees by $\tau_{assum.}$ and the dashed lines display the results of the logrank tree.

The analysis of performance measures does not indicate one CGE tree that is superior to the other ones. This result is confirmed by a visualization of the CGE‐based tree's metrics in Figure B21 in the Appendix B. In the following, we will choose the tree with $\tau_{assum.}=0.375$ for a more detailed analysis, since it provides a compromise of relatively small mean tree size, large C‐index and an IBS similar to the logrank tree's. We will compare this tree's properties to that of the logrank tree. Both trees are re‐calculated on the full dataset.

**FIGURE 5:**
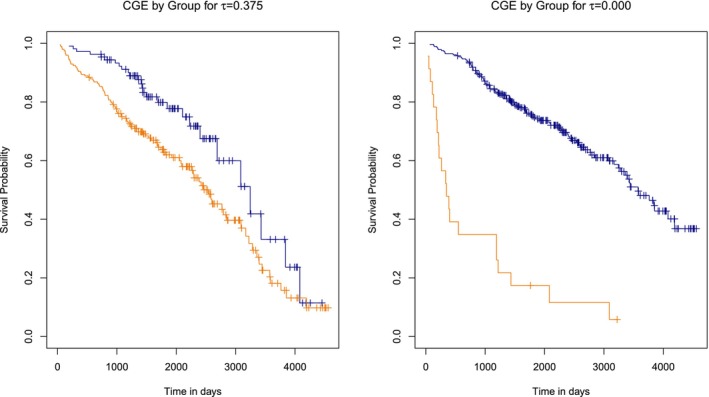
Survival curve estimates after the first split for the CGE tree with $\tau_{assum.}=0.375$ (left) and the logrank tree using the Kaplan‐Meier estimator (right).

**FIGURE B22:**
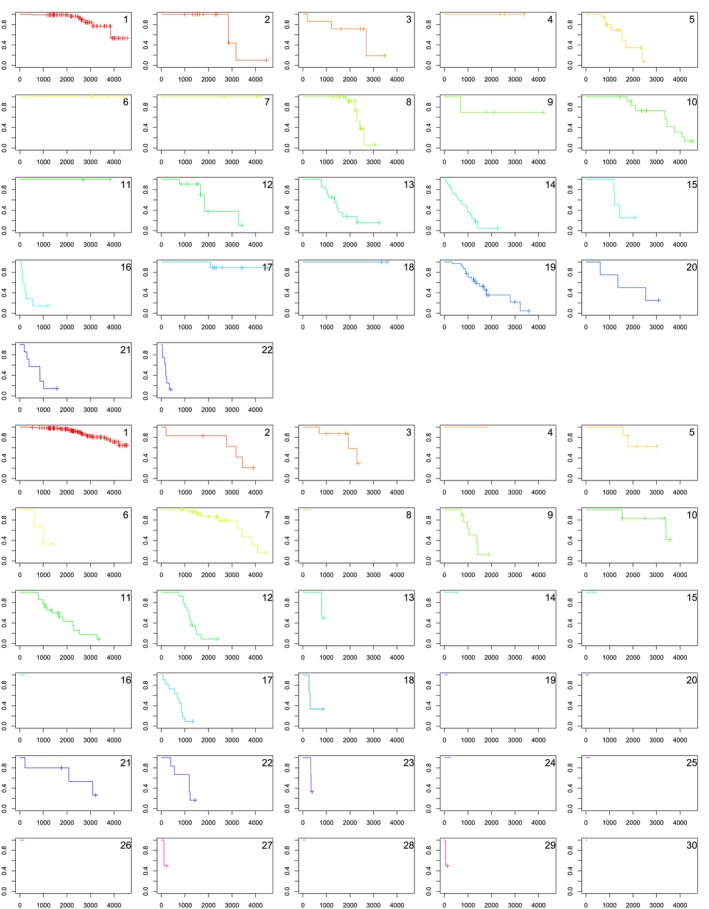
Survival curve estimates of terminal nodes, with node 1 indicating highest survival probability. CGE tree with $\tau_{assum.}=0.375$ (top) and the logrank tree using the Kaplan–Meier estimator (bottom). The trees were calculated with $\tilde{p}=0.001$. The x‐axis shows time in days, the y‐axis survival probability.

The resulting trees are again larger than the ones seen during cross validation due to increased n. This results in a CGE tree with 22 and a logrank tree with 30 terminal nodes. Both trees seem overfitted. Half of the CGE tree's terminal nodes have under five subjects. The logrank tree has 18 terminal nodes with under five and ten with only one subject. As described in Section 2.4, we ordered the tree's terminal nodes such that terminal nodes with a high number have the worst survival prognosis. However, no clear separation of the survival curves of the terminal nodes was visible and patterns in survival across terminal nodes could not be seen clearly. See Figure B22 in the Appendix B for details. Both trees use multiple variables repeatedly for splitting in subsequent splits. We thus re‐calculate both trees using a smaller p‐value threshold of $\tilde{p}=0.001$. The new trees were considerably smaller with 12 terminal nodes for the CGE tree and 19 terminal nodes for the logrank tree. We will describe these trees for the remaining part of this section:

The separate group survival curve estimates at the first split of both trees can be seen in Figure 5. The CGE tree splits the data into {Age≤45} and {Age>45}, with the first group showing longer survival. Figure 5 indeed shows a clear separation of the CGEs of the two group's survival, with the older group having the lower estimates. The first split of the logrank tree is by {Presence of Ascites>0} and {Presence of Ascites≤0}. The separated groups by the logrank test are uneven, with only 23 of 306 subjects being in the second group. Moreover, the censored subjects are distributed among groups very unevenly with only two of 183 censored subjects being in the group with lower expected survival.

**FIGURE 6:**
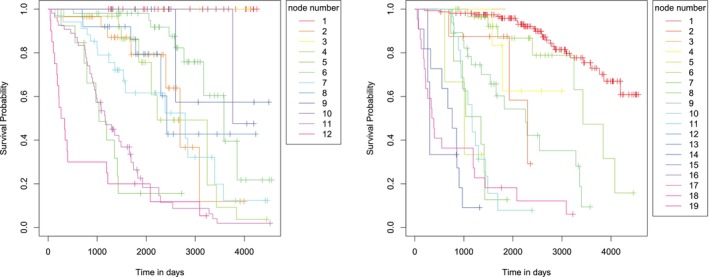
Survival curve estimates of terminal nodes, with node 1 indicating highest survival probability. CGE tree with $\tau_{assum.}=0.375$ (left) and the logrank tree using the Kaplan–Meier estimator (right). The trees were calculated with $\tilde{p}=0.001$. High node numbers indicate shorter survival.

We continue our analysis by looking at the survival curve estimators of the PBC data by terminal node in Figure 6. Still, no clear separation of estimates is visible, but there is a tendency for nodes with lower numbers to have longer survival times. The graphic for the CGE tree shows several groups that consist only of censored individuals with overlapping CGEs constantly at 1. We can also see that the terminal node sizes for the CGE tree are distributed relatively evenly by taking values between 6 and 54. The logrank tree's final node sizes differ more from each other. One terminal node contains 157, more than half, of the study subjects. Eight terminal nodes only contain one subject, and their survival estimators cannot be displayed in Figure 6. The more even distribution of subjects over terminal nodes speaks in favor of the CGE tree here.

**FIGURE 7:**
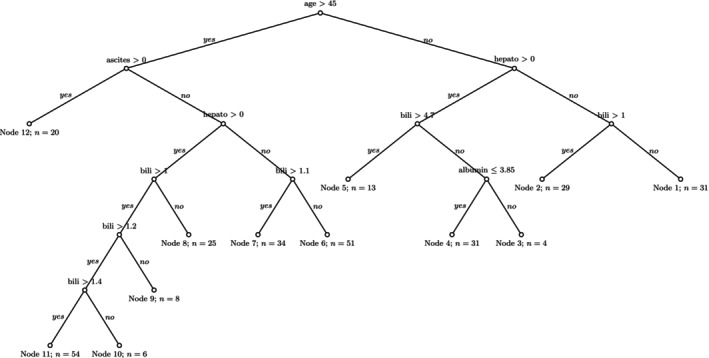
CGE survival tree with $\tau_{assum.}=0.375$ and $\tilde{p}=0.001$. High node numbers indicate shorter survival.

To end our analysis, we display the full CGE classification tree for $\tau_{assum.}=0.375$ in Figure 7. Many of the tree's splits seem reasonable, such as categorizing older patients (*Age* >45) or patients with *Presence of Hepatomegaly or Enlarged Liver* (hepto) in higher risk groups. Nine of the twelve terminal nodes have a parent node that splits according to *Serum Bilirubin*. While this points out the importance of the biomarker for liver health, it also indicates that the tree might be overfitted. In further analysis, we could, for instance, find a systematic way to summarize node 8 to 11, which result from repeatedly splitting the data according to *Serum Bilirubin*.

## 6 Discussion

In this paper, we introduced a permutation test for the null hypothesis of equal survival distributions in two groups under the assumption of equal censoring distributions. The introduced test is based on the copula‐graphic estimator, such that form and strength of a dependence between survival and censoring times can be incorporated. Assuming a Clayton copula and various dependence parameters corresponding to values of Kendall's τ between 0 and 0.75, we assessed the test's type I error and power in a simulation study. We found that the test maintains a desired type I error of α=0.05 relatively well for all considered sample sizes and censoring scenarios. Furthermore, it was robust to a misspecification of the copula model when the data was generated from a Frank copula. We were overall satisfied with the test's power, which was generally robust except in scenarios with highly unbalanced group sizes and significant censoring. Here, the logrank test demonstrated higher power than our proposed test, even under conditions of dependent censoring. The most notable power advantage of our proposed test compared to the logrank test was observed in cases with heteroscedasticity.

In practice, the problem of selecting a suitable dependence parameter $\tau_{assum.}$ for the test remains challenging. In our simulation studies, we saw that in almost all cases the power of the CGE test decreases with $\tau_{assum.}$ and the test with $\tau_{assum.}=0$ had the highest power overall. Few exceptions were seen, for instance under proportional hazards with normal covariates, varying variances across groups and high censoring (see Figure 2). However, this effect vanishes for the same setting with less censoring. Furthermore, when the assumption of equal censoring distribution across groups was violated and the censoring distribution was the same as the survival distribution, the CGE‐tests with higher $\tau_{assum.}$ performed slightly better (see Figure 4). These findings could potentially assist the search for a good $\tau_{assum.}$ and we suggest the following practical guidance: First, when the focus is predictive modeling via tree construction, $\tau_{assum.}$ can be treated as tuning parameter and selected via cross‐validation during training. Second, when inference with the CGE‐based permutation test is the primary goal, we recommend $\tau_{assum.}=0$ as the default choice. In situations with heavy (and possibly unequal) censoring and/or heteroscedasticity, a $\tau_{assum.}>0$ may be pre‐specified from domain knowledge; alternatively, results for several $\tau_{assum.}>0$ values may be reported in a sensitivity analysis without selecting one post‐hoc.

The permutation test was then implemented as a splitting criterion in a survival tree algorithm, that was tested on simulated data as well as on real‐world data from the Mayo Clinic Primary Biliary Cholangitis clinical trial. While the trees' survival predictions demonstrated satisfactory concordance in all settings, with Harrell's C‐indices exceeding 0.7, the IBS was rather high with values of over 0.28, especially for data with a high censoring rate above 46%.

We decided to only consider a single survival tree to benefit from its straightforward interpretability and low computation time. Moreover, a single survival tree provides an explicit set of decision rules that partitions patients into subgroups with distinct estimated survival curves. Such rule‐based decision paths are easier to read and communicate clinically, particularly in comparison with the random survival forest (RSF) [55], where predictions are aggregated across many trees and no single decision path exists. Nonetheless, tree ensembles such as survival forests [55] are often shown to have much higher predictive ability. Furthermore, during 10‐fold cross validation we noticed differences in the ten resulting trees, their number of terminal nodes and chosen covariates. This instability could also be reduced using an ensemble method. Although a survival forest may increase computational time and reduce interpretability, its potential predictive benefits could justify the trade‐off. Beyond the classical RSF, many other implementations exist, see for example Section 3 of Bou‐Hamad et al. (2011) [15] for an overview.

Compared with Cox‐regression, our approach avoids proportional hazards assumptions, can capture non‐linearities, and allows detection of various effect types, which can be clinically informative. On the other hand, the Cox model remains advantageous when proportional hazards is plausible: It offers direct effect size estimation and direct coefficient‐based interpretability. In a single tree, effect magnitudes are less straightforward to quantify and expressed implicitly via subgroup survival curves rather than coefficients.

### 6.1 Limitations

While we were able to implement and apply the proposed survival tree, some limitations of the method and of our simulation study should be noted:

One limitation of the proposed method is the assumption of equal censoring distributions of both groups. While this assumption (and the resulting exchangeability of survival times under the null hypothesis) is required to mathematically derive an exact test, it is not a realistic assumption and limits the use of our work on real‐world data. In the sensitivity analysis in Section 3.2.3, we found that certain violations of this assumption can degrade the test's performance. However, traditional methods such as the logrank test exhibited similar sensitivity. Other authors [38, 56] suggest an alternative permutation approach that does not require exchangeability under the null hypothesis by applying a studentization. They studentize their test statistic by adding a (co)variance estimator to the test statistic that is calculated on the same data permutations as the rest of the statistic. This studentized permutation strategy was subsequently applied to inference in factorial designs [20]. A similar studentization approach could also be derived for our statistic. Moreover, we could also think about other tests for splitting, for example, based on ideas from omnibus tests [37, 38, 56] or based on interpretable estimands like the restricted mean survival time [57].

During the evaluation of the simulation study in Section 4.2 we saw that the considered trees tend to overfit towards the training data, resulting in trees with many terminal nodes only containing a small number of training subjects and low predictive accuracy. Furthermore, this number was often larger than the number of covariates and took away some of the interpretability of our trees that we had hoped for. Currently, we address overfitting in two ways: First, we assess predictive performance out‐of‐sample on independent test data. Second, the tree growth procedure includes an explicit stopping rule (the p‐value threshold) that determines whether a split of the data into two groups is considered significant and leads to the addition of two new sub‐nodes to the tree. This regularizes tree growth and is applied consistently to ensure a fair comparison. Additional strategies against overfitting like post‐hoc pruning or imposing a minimum terminal‐node size would also be of interest. Furthermore, we could add an amalgamation step to our algorithm that reduces the number of terminal nodes by recursively grouping similar terminal nodes together [15, 58].

The logrank tree had even more terminal nodes than the copula‐graphic estimator based trees across all study settings. One reason for this is given by our p‐value threshold of 0.01 that was set for all trees before the start of the simulation study. A detailed study on its calibration for both types of trees would be necessary to properly evaluate this issue.

**FIGURE B23:**
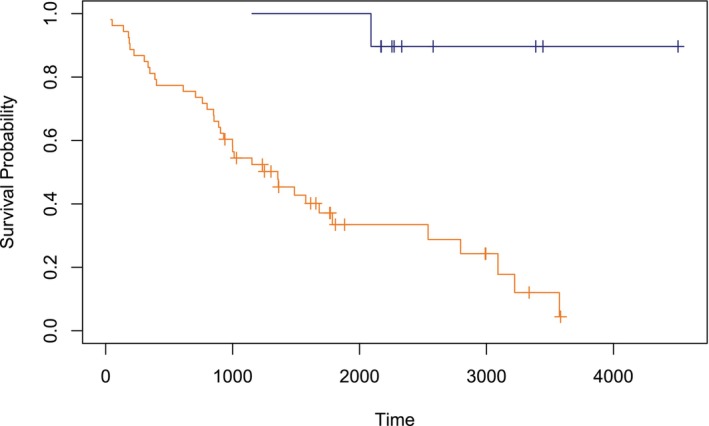
Survival curve estimates over time in days after a second split for the CGE tree with $\tau_{assum.}=0.375$.

Moreover, we saw a problem of copula‐graphic estimator trees finding splits with large absolute differences between the group's estimators by choosing one group consisting of almost exclusively censored subjects. This resulted in one group's CGE having values close to 1 over the whole study period and led to large test statistic values. To prevent splits like the one seen in Figure B23, we could add a maximum censoring threshold for each descendant group.

A further issue of the proposed permutation‐based trees was long computation time, caused by the nesting of the iterative permutation test into the iterative tree algorithm. To solve this problem, we could try to adapt a computationally efficient, matrix‐based algorithm [17] for a survival tree splitting based on score tests. While a direct transfer of this algorithm to our proposed test does not seem possible, since we use a permutation test, some modification of the proposed algorithm might be applicable for our setting.

Beyond the aforementioned limitations of the survival tree algorithm, the simulation studies and data example used to assess its performance also have limitations: During our simulation studies in Sections 3 and 4 we generated survival data with two different censoring‐event dependencies, using a Clayton and a Frank copula. Exploring a broader range of copulas is an interesting extension. For instance, considering copula families with strong upper‐tail dependence (e.g., the Gumbel copula in case of late events) could potentially lead to different empirical split patterns.

In our simulation study assessing the performance of CGE trees on simulated data in Section 4, we generated survival times depending on blocks of informative and non‐informative covariates. We restricted pairwise interaction to covariates within the same block, to mimic a pathway of genes. Since real‐world data may exhibit more complex interaction patterns (including interactions spanning across blocks), we acknowledge that our simulation setting is limited in this respect. While this may affect the absolute performance of specific models, our primary goal was an initial, controlled comparison of the tree algorithm under a structured pathway‐like setting, such that the results remain informative for relative method comparison. Nevertheless, further studies with more complex covariate patterns would be valuable.

Lastly, this paper only considers one real‐world clinical data example. While this case study provides a first illustration of the method's applicability, a multi‐dataset benchmarking study is another important direction for future work.

## Funding

The authors have nothing to report.

## Disclosure

The authors have nothing to report.

## Conflicts of Interest

The authors declare no conflicts of interest.

## Figures and Tables

**FIGURE C1:**
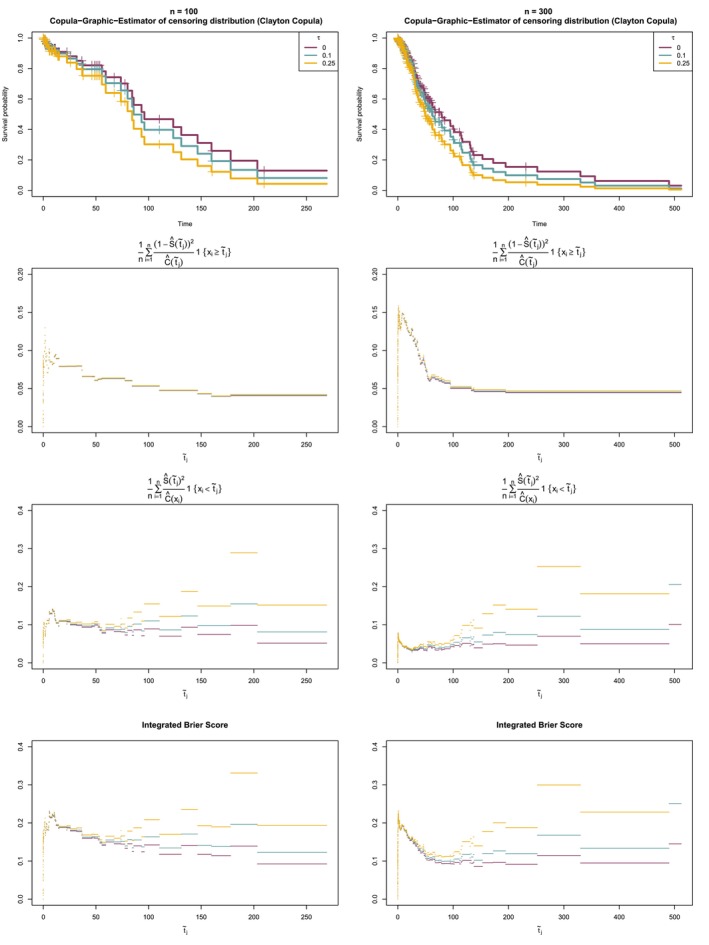
Decomposition of the IBS calculated for τ∈{0,0.1,0.25}, for a CGE‐tree with $\tau_{assum.}=0.25$ and n=100 (left column) and n=300 (right column).

## Data Availability

The data that support the findings of this study are available in CRAN at https://cran.r‐project.org/. These data were derived from the following resources available in the public domain:‐R package: survival: Survival Analysis, https://cran.r‐project.org/web/packages/survival/index.html.

## ENDNOTES

^1^
The first entry of $\theta_{assum.}$ is in fact 0.00020002 and that of $\tau_{assum.}$ is 0.0001. They were chosen slightly larger than 0 to use the same algorithms as for larger τ.

^2^

R code for the estimator $\hat{IB}$ is taken from the package SurvMetrics [52], where Kaplan‐Meier estimators were replaced with CGEs. Instead of considering step‐functions, SurvMetrics interpolates the Kaplan‐Meier estimate for the censoring function in between censoring times (see code of function Gt() for details). In this paper, all estimators are step functions. Thus, our estimate of IB for $\tau_{theor.}\approx 0$ is only identical to SurvMetrics's estimate, if the $\tilde{t}$ are exactly the timepoints where a censoring was observed. However, the deviations between the methods are negligibly small.

## Appendix A Tables

**TABLE A1:** Type I error rate estimates (empirical standard errors) for $n_{1}=n_{2}=50$ by assumed dependency parameter $\tau_{theor.}$ on Clayton copula generated data. The simulations were performed with $n_{\text{perm}}=5000$ permutations for the permutation test on $n_{\text{sim}}=5000$ generated datasets per setting. This table corresponds to Table A3.

		Copula‐graphic estimator permutation test											
$\tau_{theor.}$	cens. %	$\tau_{assum.}=0$		$\tau_{assum.}=0.25$		$\tau_{assum.}=0.5$		$\tau_{assum.}=0.75$		Logrank test		Peto–Peto test	
0	0.100	0.050	(0.003)	0.053	(0.003)	0.056	(0.003)	0.056	(0.003)	0.056	(0.003)	0.052	(0.003)
	0.250	0.052	(0.003)	0.049	(0.003)	0.050	(0.003)	0.051	(0.003)	0.056	(0.003)	0.049	(0.003)
	0.499	0.046	(0.003)	0.050	(0.003)	0.052	(0.003)	0.049	(0.003)	0.048	(0.003)	0.043	(0.003)
0.25	0.066	0.056	(0.003)	0.054	(0.003)	0.055	(0.003)	0.054	(0.003)	0.056	(0.003)	0.057	(0.003)
	0.191	0.047	(0.003)	0.047	(0.003)	0.048	(0.003)	0.049	(0.003)	0.051	(0.003)	0.048	(0.003)
	0.500	0.050	(0.003)	0.047	(0.003)	0.049	(0.003)	0.050	(0.003)	0.048	(0.003)	0.051	(0.003)
0.5	0.038	0.052	(0.003)	0.055	(0.003)	0.055	(0.003)	0.055	(0.003)	0.057	(0.003)	0.052	(0.003)
	0.124	0.049	(0.003)	0.051	(0.003)	0.051	(0.003)	0.048	(0.003)	0.051	(0.003)	0.051	(0.003)
	0.500	0.051	(0.003)	0.052	(0.003)	0.047	(0.003)	0.047	(0.003)	0.051	(0.003)	0.045	(0.003)
0.75	0.017	0.049	(0.003)	0.050	(0.003)	0.049	(0.003)	0.050	(0.003)	0.053	(0.003)	0.047	(0.003)
	0.061	0.055	(0.003)	0.056	(0.003)	0.056	(0.003)	0.056	(0.003)	0.064	(0.003)	0.055	(0.003)
	0.501	0.051	(0.003)	0.049	(0.003)	0.049	(0.003)	0.049	(0.003)	0.049	(0.003)	0.047	(0.003)

*Note:* The empirical standard error refers to the uncertainty caused by performing the simulation study with a finite number of simulations. See Section 3.1 for details.

**TABLE A2:** Type I error rate estimates (empirical standard errors) for $n_{1}=n_{2}=20$ by assumed dependency parameter $\tau_{theor.}$ on Clayton copula generated data. The simulations were performed with $n_{\text{perm}}=5000$ permutations for the permutation test on $n_{\text{sim}}=5000$ generated datasets per setting. This table corresponds to Table A4.

		Copula‐graphic estimator permutation test											
$\tau_{theor.}$	cens. %	$\tau_{assum.}=0$		$\tau_{assum.}=0.25$		$\tau_{assum.}=0.5$		$\tau_{assum.}=0.75$		Logrank test		Peto–Peto test	
0	0.099	0.051	(0.003)	0.053	(0.003)	0.051	(0.003)	0.052	(0.003)	0.060	(0.003)	0.057	(0.003)
	0.250	0.048	(0.003)	0.046	(0.003)	0.047	(0.003)	0.050	(0.003)	0.056	(0.003)	0.048	(0.003)
	0.501	0.052	(0.003)	0.051	(0.003)	0.049	(0.003)	0.050	(0.003)	0.055	(0.003)	0.055	(0.003)
0.25	0.065	0.050	(0.003)	0.049	(0.003)	0.048	(0.003)	0.049	(0.003)	0.059	(0.003)	0.050	(0.003)
	0.192	0.047	(0.003)	0.045	(0.003)	0.045	(0.003)	0.045	(0.003)	0.052	(0.003)	0.046	(0.003)
	0.500	0.050	(0.003)	0.050	(0.003)	0.051	(0.003)	0.052	(0.003)	0.060	(0.003)	0.054	(0.003)
0.5	0.038	0.049	(0.003)	0.048	(0.003)	0.049	(0.003)	0.046	(0.003)	0.055	(0.003)	0.050	(0.003)
	0.126	0.049	(0.003)	0.050	(0.003)	0.053	(0.003)	0.052	(0.003)	0.058	(0.003)	0.057	(0.003)
	0.501	0.051	(0.003)	0.050	(0.003)	0.050	(0.003)	0.048	(0.003)	0.052	(0.003)	0.053	(0.003)
0.75	0.017	0.050	(0.003)	0.050	(0.003)	0.049	(0.003)	0.048	(0.003)	0.054	(0.003)	0.052	(0.003)
	0.060	0.051	(0.003)	0.051	(0.003)	0.051	(0.003)	0.049	(0.003)	0.057	(0.003)	0.051	(0.003)
	0.501	0.053	(0.003)	0.053	(0.003)	0.056	(0.003)	0.053	(0.003)	0.060	(0.003)	0.057	(0.003)

*Note:* The empirical standard error refers to the uncertainty caused by performing the simulation study with a finite number of simulations. See Section 3.1 for details.

**TABLE A3:** Type I error rate estimates (empirical standard errors) for $n_{1}=n_{2}=50$ by assumed dependency parameter $\tau_{theor.}$ on Clayton copula generated data. This table was re‐created as part of the sensitivity analysis with $n_{sim}=n_{perm}=5000$. See Table A1 for results.

		Copula‐graphic estimator permutation test									
$\tau_{theor.}$	cens. %	$\tau_{assum.}=0$		$\tau_{assum.}=0.25$		$\tau_{assum.}=0.5$		$\tau_{assum.}=0.75$		Logrank test	
0	0.100	0.044	(0.005)	0.043	(0.005)	0.040	(0.004)	0.043	(0.005)	0.048	(0.005)
	0.250	0.051	(0.005)	0.054	(0.005)	0.056	(0.005)	0.052	(0.005)	0.053	(0.005)
	0.500	0.041	(0.004)	0.047	(0.005)	0.047	(0.005)	0.052	(0.005)	0.046	(0.005)
0.25	0.066	0.048	(0.005)	0.048	(0.005)	0.051	(0.005)	0.048	(0.005)	0.054	(0.005)
	0.192	0.050	(0.005)	0.046	(0.005)	0.048	(0.005)	0.046	(0.005)	0.056	(0.005)
	0.501	0.051	(0.005)	0.047	(0.005)	0.044	(0.005)	0.046	(0.005)	0.055	(0.005)
0.5	0.038	0.048	(0.005)	0.048	(0.005)	0.051	(0.005)	0.052	(0.005)	0.054	(0.005)
	0.123	0.054	(0.005)	0.055	(0.005)	0.055	(0.005)	0.054	(0.005)	0.055	(0.005)
	0.501	0.050	(0.005)	0.052	(0.005)	0.047	(0.005)	0.047	(0.005)	0.049	(0.005)
0.75	0.017	0.049	(0.005)	0.051	(0.005)	0.051	(0.005)	0.048	(0.005)	0.048	(0.005)
	0.060	0.048	(0.005)	0.051	(0.005)	0.052	(0.005)	0.054	(0.005)	0.055	(0.005)
	0.499	0.051	(0.005)	0.048	(0.005)	0.048	(0.005)	0.048	(0.005)	0.052	(0.005)

*Note:* The empirical standard error refers to the uncertainty caused by performing the simulation study with a finite number of simulations. See Section 3.1 for details.

**TABLE A4:** Type I error rate estimates (empirical standard errors) for $n_{1}=n_{2}=20$ by assumed dependency parameter $\tau_{theor.}$ on Clayton copula generated data. This table was re‐created as part of the sensitivity analysis with $n_{sim}=n_{perm}=5000$. See Table A2 for results.

		Copula‐graphic estimator permutation test									
$\tau_{theor.}$	cens. %	$\tau_{assum.}=0$		$\tau_{assum.}=0.25$		$\tau_{assum.}=0.5$		$\tau_{assum.}=0.75$		Logrank test	
0	0.100	0.040	(0.004)	0.043	(0.005)	0.046	(0.005)	0.041	(0.004)	0.054	(0.005)
	0.247	0.054	(0.005)	0.056	(0.005)	0.055	(0.005)	0.056	(0.005)	0.057	(0.005)
	0.499	0.050	(0.005)	0.046	(0.005)	0.048	(0.005)	0.046	(0.005)	0.059	(0.005)
0.25	0.065	0.052	(0.005)	0.046	(0.005)	0.046	(0.005)	0.046	(0.005)	0.057	(0.005)
	0.192	0.053	(0.005)	0.046	(0.005)	0.046	(0.005)	0.046	(0.005)	0.060	(0.005)
	0.498	0.051	(0.005)	0.050	(0.005)	0.046	(0.005)	0.051	(0.005)	0.052	(0.005)
0.5	0.038	0.056	(0.005)	0.054	(0.005)	0.054	(0.005)	0.053	(0.005)	0.064	(0.005)
	0.126	0.048	(0.005)	0.045	(0.005)	0.048	(0.005)	0.049	(0.005)	0.052	(0.005)
	0.502	0.058	(0.005)	0.060	(0.005)	0.058	(0.005)	0.059	(0.005)	0.058	(0.005)
0.75	0.017	0.061	(0.005)	0.060	(0.005)	0.060	(0.005)	0.060	(0.005)	0.067	(0.006)
	0.059	0.051	(0.005)	0.051	(0.005)	0.054	(0.005)	0.052	(0.005)	0.062	(0.005)
	0.500	0.048	(0.005)	0.050	(0.005)	0.053	(0.005)	0.052	(0.005)	0.054	(0.005)

*Note:* The empirical standard error refers to the uncertainty caused by performing the simulation study with a finite number of simulations. See Section 3.1 for details.

**TABLE A5:** Type I error rate estimates (empirical standard errors) for $n_{1}=n_{2}=200$ by assumed dependency parameter $\tau_{theor.}$ on Clayton copula generated data.

		Copula‐graphic estimator permutation test									
$\tau_{theor.}$	cens. %	$\tau_{assum.}=0$		$\tau_{assum.}=0.25$		$\tau_{assum.}=0.5$		$\tau_{assum.}=0.75$		Logrank test	
0	0.100	0.051	(0.005)	0.045	(0.005)	0.047	(0.005)	0.049	(0.005)	0.047	(0.005)
	0.250	0.043	(0.005)	0.043	(0.005)	0.046	(0.005)	0.046	(0.005)	0.050	(0.005)
	0.500	0.048	(0.005)	0.052	(0.005)	0.054	(0.005)	0.058	(0.005)	0.053	(0.005)
0.25	0.066	0.047	(0.005)	0.048	(0.005)	0.049	(0.005)	0.050	(0.005)	0.048	(0.005)
	0.191	0.056	(0.005)	0.057	(0.005)	0.056	(0.005)	0.056	(0.005)	0.059	(0.005)
	0.501	0.052	(0.005)	0.057	(0.005)	0.056	(0.005)	0.056	(0.005)	0.050	(0.005)
0.5	0.039	0.057	(0.005)	0.056	(0.005)	0.054	(0.005)	0.057	(0.005)	0.059	(0.005)
	0.124	0.043	(0.005)	0.047	(0.005)	0.046	(0.005)	0.046	(0.005)	0.047	(0.005)
	0.500	0.049	(0.005)	0.045	(0.005)	0.046	(0.005)	0.041	(0.004)	0.049	(0.005)
0.75	0.017	0.052	(0.005)	0.054	(0.005)	0.053	(0.005)	0.055	(0.005)	0.049	(0.005)
	0.060	0.053	(0.005)	0.052	(0.005)	0.052	(0.005)	0.055	(0.005)	0.057	(0.005)
	0.500	0.049	(0.005)	0.047	(0.005)	0.052	(0.005)	0.056	(0.005)	0.051	(0.005)

*Note:* The empirical standard error refers to the uncertainty caused by performing the simulation study with a finite number of simulations. See Section 3.1 for details.

**TABLE A6:** Exemplary mean censoring proportions of $n_{sim}=5000$ datasets for $n_{1}=n_{2}=150$, $\tau_{theor.}=0.5$ for the Clayton copula and r=0.5 by β and group.

β	−1.400	−1.000	−0.600	−0.400	−0.200	0.000	0.200	0.400	0.600	1.000	1.400
Group 1	0.501	0.500	0.501	0.501	0.499	0.500	0.499	0.501	0.499	0.501	0.499
Group 2	0.703	0.679	0.638	0.604	0.558	0.508	0.435	0.386	0.356	0.327	0.297

**TABLE A7:** Type I error rate estimates (empirical standard errors) for $n_{1}=n_{2}=150$ by assumed dependency parameter $\tau_{theor.}$ on Clayton copula generated data. The simulations were performed with $n_{\text{perm}}=1000$ permutations for the permutation test on $n_{\text{sim}}=1000$ generated datasets per setting. Data was generated using a Gompertz model as described above with $\beta_{1}=\beta_{2}=0$.

		Copula‐graphic estimator permutation test											
$\tau_{theor.}$	cens. %	$\tau_{assum.}=0$		$\tau_{assum.}=0.25$		$\tau_{assum.}=0.5$		$\tau_{assum.}=0.75$		Logrank test		Peto–Peto test	
0	0.063	0.057	(0.007)	0.052	(0.007)	0.052	(0.007)	0.052	(0.007)	0.053	(0.007)	0.048	(0.007)
	0.403	0.044	(0.006)	0.042	(0.006)	0.049	(0.007)	0.048	(0.007)	0.047	(0.007)	0.051	(0.007)
0.5	0.030	0.040	(0.006)	0.040	(0.006)	0.043	(0.006)	0.038	(0.006)	0.044	(0.006)	0.039	(0.006)
	0.303	0.050	(0.007)	0.040	(0.006)	0.041	(0.006)	0.043	(0.006)	0.052	(0.007)	0.047	(0.007)
0.95	0.003	0.046	(0.007)	0.048	(0.007)	0.050	(0.007)	0.050	(0.007)	0.052	(0.007)	0.052	(0.007)
	0.108	0.048	(0.007)	0.053	(0.007)	0.048	(0.007)	0.040	(0.006)	0.059	(0.007)	0.044	(0.006)

*Note:* The empirical standard error refers to the uncertainty caused by performing the simulation study with a finite number of simulations. See Section 3.1 for details.

## Appendix C CGE‐Based Integrated Brier Score

The Integrated Brier Score (IBS) is calculated as

$$\begin{array}{ll} \hat{IB} & =\frac{1}{\max(x)}\sum_{j=1}^{m-1}\left\{({\tilde{t}}_{j+1}-{\tilde{t}}_{j})\frac{1}{n}\sum_{i=1}^{n}\left\{\frac{{\left(1-\hat{S}({\tilde{t}}_{j}|z_{i})\right)}^{2}}{\hat{C}({\tilde{t}}_{j})}1\{x_{i}\geq \tilde{t_{j}}\}+\frac{\hat{S}{\left({\tilde{t}}_{j}|z_{i}\right)}^{2}}{\hat{C}(x_{i})}1\{x_{i}<\tilde{t_{j}}\}\right\}\right\}. \end{array}$$

For a given tree, the estimates of the survival functions Ŝ are identical for the traditional IBS and for our CGE‐adjusted IBS, since they depend only on the Ŝ produced by the tree. Therefore, the only difference between the two scores lies in the estimation of the censoring distribution used to weight the survival estimates, denoted by Ĉ(·). This estimate is either computed with the CGE (for some τ>0), or the Kaplan–Meier estimator which corresponds to the case τ=0.

As illustrated in Figure B1 of the paper, the CGE of the survival function, for any dataset at a given time point, decreases as τ increases. This is because larger values of τ correspond to the assumption that censoring is more informative on the survival probabilities, and observed censorings result in a larger decline of the CGE. The same behavior applies to the CGE of the censoring function, with the roles of observed events and censorings interchanged.

Therefore, for larger values of τ, the estimated censoring weights Ĉ appearing in the denominator of the IBS are smaller, which leads to an increase in the overall score. This behavior is illustrated in Figure C1, where the IBS is plotted for an exemplary CGE tree with n=100 (left column) and n=300 (right column) for τ∈{0,0.1,0.25}, with τ=0 corresponding to the regular IBS.

The first row of Figure C1 shows the censoring estimators Ĉ for τ∈{0,0.1,0.25}. These estimators mainly differ toward the end of the study period, after many events have already been observed and weighted as more or less informative depending on the choice of $\tau_{theor}$. Meanwhile, the time between observed events increases toward the end of the study, as most events occur early and only a few individuals survive for longer durations.

The IBS integrates a step function that is weighted by the censoring estimator Ĉ and has jump points at the same values that Ĉ does. At any time point ${\tilde{t}}_{j}$, this integral can be decomposed into two sums: one corresponding to observations before ${\tilde{t}}_{j}$ (see the second row of Figure C1) and one corresponding to observations after ${\tilde{t}}_{j}$ (see the third row).

Toward the end of the study period, only a small number of subjects contribute to the second component, that is, to observations occurring after ${\tilde{t}}_{j}$. Furthermore, the corresponding step function remains constant over increasingly long time intervals, reflecting the sparsity of events in this late part of the study.

At this stage, differences in the censoring estimator Ĉ induced by the choice of $\tau_{theor.}$ (which are most pronounced toward the end of the study) can therefore have a substantial impact on the overall IBS. This effect is illustrated in rows two to four of Figure C1, which display the full IBS as well as its two components associated with observations before and after the time point ${\tilde{t}}_{j}$.

For large sample sizes n, more individuals survive to later time points, effectively extending the study period and amplifying this effect. For the Kaplan–Meier‐based IBS, normalization by max(X) reduces these effects, but under the CGE weighting the late‐time contributions can still dominate because Ĉ becomes smaller for larger τ and the IBS increases.

If one nevertheless chooses to use the CGE‐based IBS, because Kaplan–Meier‐based estimation of the censoring distribution is considered too optimistic, the resulting values may be less directly comparable across different sample size settings (e.g., n=100 versus n=300). Nevertheless, comparisons remain feasible within the same setting, allowing relative performance assessment across survival tree algorithms.
